# The health and economic burden of podoconiosis in East Africa: A systematic review and meta-analysis of health outcomes with narrative synthesis of economic evidence

**DOI:** 10.1371/journal.pntd.0014427

**Published:** 2026-06-17

**Authors:** Nadia Hitimana, Vasso Anagnostopoulou, Stephen Bremner, Naillah Umutoni Uwimana, Natalia Hounsome, Lawrence Rugema, Leon Mutesa, Maya Semrau

**Affiliations:** 1 Centre for Equitable Global Health Research, Brighton and Sussex Medical School, Falmer, Brighton, United Kingdom; 2 Department of Primary Care and Public Health, Brighton and Sussex Medical School, Falmer, Brighton, United Kingdom; 3 Edinburg Napier University, Edinburgh, United Kingdom; 4 University of Rwanda, Department of Community Health, School of Public Health, College of Medicine and Health Sciences, Kigali, Rwanda; 5 University of Rwanda, Center for Human Genetics, College of Medicine and Health Sciences, Kigali, Rwanda; Consejo Nacional de Investigaciones Cientificas y Tecnicas, Fundación Mundo Sano, ARGENTINA

## Abstract

**Background:**

Podoconiosis is a neglected tropical disease (NTD) causing chronic lower limb lymphoedema through prolonged barefoot exposure to irritant volcanic soils. Despite affecting an estimated 4 million people globally, podoconiosis remains absent from the Global Burden of Disease study and severely under-researched relative to its impact. East Africa carries the greatest regional burden, yet no comprehensive synthesis of prevalence, disability-adjusted life years (DALYs), or economic burden data exists for the region. This systematic review and meta-analysis aimed to address this gap by synthesizing available evidence on the prevalence and health and economic burden of podoconiosis across East Africa.

**Methods:**

We conducted a systematic search of PubMed/MEDLINE, EMBASE, SCOPUS, Web of Science, EconLit, WHO AFROLIB, and Google Scholar for studies published in English between 2011 and 2023. Studies reporting population-based podoconiosis prevalence, DALYs, or economic burden data from East African countries as defined by the UN M49 geoscheme were eligible for inclusion. Grey literature was also searched, including reports, ministry of health documents, and conference proceedings both in English and French, to capture evidence not indexed in academic databases. The search was updated in March 2026 to ensure the most current evidence was captured. Quality assessment was performed using the Newcastle-Ottawa Scale. Prevalence data were pooled using a random-effects model with the DerSimonian-Laird estimator and Freeman-Tukey double arcsine transformation. Subgroup analyses were conducted by country, sample size, and geographic scope.

**Results:**

Fourteen studies met the inclusion criteria, comprising 10 from Ethiopia, 2 from Kenya, and 1 each from Uganda and Rwanda, collectively examining 1,720,437 individuals. The overall pooled prevalence was 1.19 (95% CI: 1.14–1.25) on the transformed scale, with extreme heterogeneity (I² = 99.0%). The Ethiopia-specific pooled prevalence was 4.52% (95% CI: 3.92–5.16%), compared to 0.20% (95% CI: 0.10–0.33%) for the non-Ethiopian subgroup, a more than 20-fold difference. A consistent inverse relationship between sample size and observed prevalence was identified across all analyses, reflecting the systematic tendency of smaller studies to target confirmed endemic foci while larger surveys captured broader populations. Only one study Deribe et al. (2020), reported DALY and economic burden estimates, confined to Ethiopia, estimating 172,073 DALYs annually and a total economic burden of US$213.2 million per year.

**Conclusion:**

Podoconiosis imposes a substantial but profoundly under-quantified burden across East Africa, with disease intensity disproportionately concentrated in Ethiopian highland communities. The near-complete absence of DALY and economic burden data outside Ethiopia represents a critical evidence gap. Standardised nationally representative surveys, expansion of burden modelling beyond Ethiopia, and advocacy for inclusion of podoconiosis in the Global Burden of Disease study are urgently needed to support evidence-based policy and resource prioritisation across the region. This systematic review was registered in the PROSPERO International Prospective Register of Systematic Reviews with the registration number: CRD42023432640.

## Author summary

Podoconiosis is a preventable disease that causes severe and painful swelling of the lower legs, affecting millions of people living in poverty in tropical highland regions who walk barefoot on volcanic soils. Despite being recognized as a neglected tropical disease by the World Health Organization since 2011, podoconiosis remains largely invisible in global health accounting; it is not included in the Global Burden of Disease study and receives minimal research funding. We conducted a systematic review and meta-analysis on the prevalence of podoconiosis in East Africa and what it costs in terms of health and economic burden. We found 14 studies from four countries—Ethiopia, Kenya, Uganda, and Rwanda—but the evidence was heavily concentrated in Ethiopia, which contributed 10 of the 14 studies. Podoconiosis was far more common in Ethiopian highland communities than elsewhere. We also found that only one study had attempted to quantify the disease’s broader health and economic burden, and that study was limited to Ethiopia alone. This means that for millions of people living at risk of podoconiosis in Kenya, Uganda, Rwanda, and beyond, we simply do not know the full scale of what this disease costs in lost years of healthy life or in economic productivity. Our findings make a case that podoconiosis deserves more research attention, better funded surveillance systems, and a place in the global frameworks that determine how health resources are allocated.

## Introduction

Podoconiosis is a neglected tropical disease (NTD) that primarily affects individuals living in impoverished communities [1]. It is mainly found in tropical Africa, Central and South America, and Southeast Asia and affects an estimated 4 million people globally [2]. Podoconiosis is a condition that is often overlooked and can be mistaken for another disease known as lymphatic filariasis.

Podoconiosis is characterized by persistent swelling and inflammation (lymphoedema) of the lower limbs, which typically results from long-term barefoot exposure to irritating volcanic soils [1]. The disease can lead to physical limitations, reduced mental well-being, social stigma, and an inability to work, placing a significant social and economic burden on affected individuals and their families in underserved communities [3]. The disease’s chronic nature often results in bacterial infections, ulcers, and stiff toes, which can be debilitating and lead to lost productivity over the years. Most people develop symptoms in their second or third decade, and its prevalence continues to rise until their sixth decade [4]. If left untreated, the swelling caused by podoconiosis can worsen, leading to deformities that make it even more difficult for individuals to walk and perform daily activities [4]. The occurrence of acute dermatolymphangioadenitis(ADLA) is particularly severe among patients with lymphoedema, and it is believed to have similar causes in those with podoconiosis as with other types of lymphoedema [5]. These episodes frequently occur, and they range from 5 to 23 per year, significantly contributing to the disability and social consequences of the disease [6]. Recent randomized controlled trials have demonstrated that simple hygiene-based treatments are effective in managing podoconiosis, with one such trial in Ethiopia showing a 20% reduction in the incidence of ADLA [7].

A total of 32 countries, both current and historical, have reported cases of podoconiosis globally. Previous investigations have estimated the global prevalence of podoconiosis to range between 0.10% and 8.08% [2]. In Africa, 18 countries have been identified as having people affected by podoconiosis [8]. Among these African countries, podoconiosis has been reported mainly in the East and West African regions [9]. High prevalence rates were observed in Cameroon (8.08%), Ethiopia (7.45%), Uganda (4.52%), Kenya (3.87%), and Tanzania (2.51%) reporting the highest prevalence compared to other countries [10–14] [15].

It is important to note that some of the data from these African countries comes from the 1970s and even before that (in the case of Tanzania), making it unclear what the current situation is [16]. In addition, based on misdiagnosis of podoconiosis in the past, it is most likely that the disease was confused with other causes of lymphedoema previously and hence underreported.

A predictive model assessed the environmental suitability for podoconiosis across numerous regions in Africa, with the most predictions concentrated primarily in the East African region [17]. The model identified suitability in 29 countries, distributed as follows: East [11], Central [5], West [5], North [4], and South [4]. Utilizing remote sensing techniques, efforts have also been made to identify and estimate areas and populations at risk of podoconiosis by considering climate and ecological indicators [17]. As of 2020, it was estimated that more than 114.5 million people resided in regions environmentally conducive to podoconiosis [17]. The predominant portion of this population, accounting for 81.7%, was located in East Africa [17]. Specifically, Ethiopia, Uganda, Rwanda, and Burundi were identified as countries with predicted suitability for the occurrence of podoconiosis in that region [17].

Several NTDs, such as podoconiosis, do not result in fatalities; however, they do cause disability and disfigurement and can even impede the cognitive development of children. In sub-Saharan Africa, NTDs collectively contribute to approximately 534,000 deaths annually, and an estimated 57 million Disability-Adjusted Life-Years (DALYs) are lost each year due to these diseases [18]. The health, social, and financial burdens imposed by NTDs significantly affect both households and governments. These burdens stem from the direct costs of care and productivity losses arising from disability and morbidity. The impact of NTDs is predominantly felt in terms of morbidity rather than mortality. In 2012, NTDs accounted for roughly 22 million DALYs globally, representing around 40 percent of the DALYs for malaria and about 1 percent of the global total [19]. This burden is particularly significant in regions and countries where NTDs are most prevalent, with several Sub-Saharan African countries experiencing NTDs constituting more than 6 percent of the total disease burden [20].

In recent years, there have been notable advancements in understanding and managing podoconiosis. Despite these strides, further research is essential to formulate a comprehensive global control strategy [21]. A key research priority should focus on a better understanding of the global burden of podoconiosis through mapping and research. This approach will facilitate the identification of the true prevalence of the disease, enabling targeted and cost-effective interventions in Africa. To date, only three countries (Cameroon, Ethiopia, and Rwanda) have conducted nationwide mapping of podoconiosis as part of developing a global atlas [22]. However, these data are insufficient to convincingly demonstrate the extent of the podoconiosis burden for resource commitment by donors or to provide ministries of health and policymakers with the necessary data for intervention planning [23].

Podoconiosis is a disease with substantial and quantifiable burden that remains among the most neglected even within the NTD category. Although the World Health Organization included podoconiosis on its list of NTDs in 2011, its global distribution remains poorly understood, and almost all available prevalence data originate from the African region (Deribe et al., 2018). This epidemiological invisibility is compounded by a critical structural deficit in that podoconiosis has not been incorporated into the Global Burden of Disease (GBD) study, the world’s most authoritative framework for comparing disease burden across conditions, and so advocacy to include it has been identified as a priority to generate the robust global estimates needed to advance elimination efforts and support evidence-based resource allocation (Davey et al., 2018). The burden of this disease has mostly been attributable to chronic lymphoedema rather than acute episodes (Deribe et al., 2020). By contrast, comparator NTDs within the GBD framework carry substantially larger recognized global burdens: in 2015, soil-transmitted helminthiases accounted for approximately 4,443 thousand DALYs globally, schistosomiasis for 3,514 thousand, dengue fever for 2,610 thousand, and lymphatic filariasis for 2,071 thousand DALYs (Lozano et al., 2018). While podoconiosis cannot be directly compared to these figures given its absence from the GBD, the scale of the disparity points strongly to systematic underestimation of its burden rather than genuinely lower disease impact.

The 2023 global report on NTDs set a target of a 75% reduction in DALYs related to NTDs by 2030 compared to 2020. However, as of 2019 data from 181 countries, the percentage reduction in NTD DALYs between 2015 and 2019 was only 11%, based on available GBD data for 16 NTDs. Notably, the report lacked health burden estimates for various other NTDs, including podoconiosis [24]. Accurate data on the global burden and distribution of podoconiosis are crucial for the successful advocacy of including podoconiosis in Global Burden of Disease (GBD) estimations. Generating this data will also contribute towards advancing the goal of elimination and serving as a tool for evidence-based advocacy [23].

In this study, we conducted a review of the existing literature to assess the health burden of podoconiosis in the East African region, considering both prevalence and DALYs, which account for years of life lived with disability. Additionally, we synthesized the available literature on the economic burden of podoconiosis within the same geographical area.

### Objectives

Understanding the relationship between prevalence and DALYs for podoconiosis is crucial to aid in guiding public health interventions and facilitating evidence-based decision-making in healthcare for affected populations. With this systematic review, we aimed to summarize and present evidence (data) in East Africa on:

- The prevalence of podoconiosis in the region and,
- The absolute measures of disability associated with podoconiosis, i.e., DALYs and their attributed economic burden including productivity loss.

## Methods and materials

### Database searches

**Fig 1:**
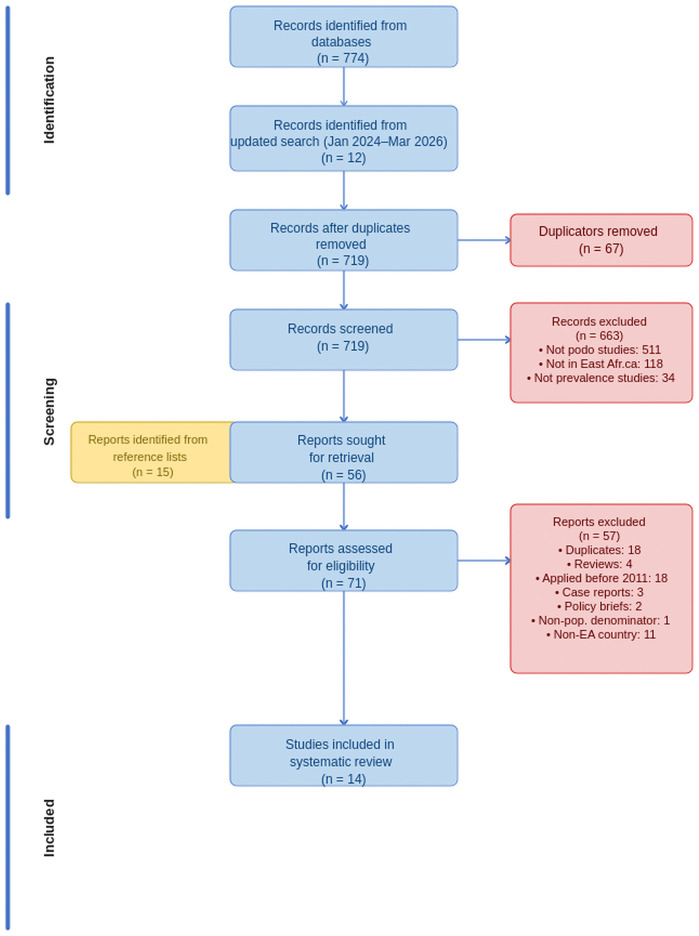
PRISMA 2020 flow diagram: Podocononiosis prevalence studies.

This systematic review searched for studies that reported the prevalence of podoconiosis and cost per podoconiosis DALY following the Preferred Reporting Items for Systematic Reviews and Meta-Analyses (PRISMA) [25], as shown in Fig 1.

We conducted searches from October 2023 to January 2024 in PubMed/MEDLINE, EMBASE, SCOPUS, Web of Science, EconLit, WHO (World Health Organization) AFROLIB, and Google Scholar for all articles published in English from 2011 to 2023. To ensure currency of the evidence, an updated search was conducted in March 2026 across the same databases, covering the period from January 2024 to March 2026. We used the following search terms: ‘podoconiosis’ OR ‘mossy foot’ OR ‘non-filarial elephantiasis’ combined (using the Boolean operator ‘AND’) with ‘prevalence’ OR ‘epidemiology’ OR ‘health burden’ OR ‘public health’, OR ‘population’ OR ‘economic burden’ OR ‘economic impact’ OR ‘DALYs.’

The review included economic evaluation studies, both trial-based and modeled, which considered cost analysis, cost-benefit analysis, cost-utility analysis, and cost-effectiveness analysis. We searched for studies with the types of outcomes of interest, including cost–benefit measurements such as incremental cost-effectiveness ratio (ICER) and incremental cost per disability-adjusted life year (DALY). We limited our search to the East African region. Reference lists of all included studies were manually searched to identify any additional relevant articles that may have been missed. Through reading abstracts, full articles were retrieved as they appeared to meet the inclusion criteria. Case reports and reviews were not included in this review.

The geographical scope of this review was defined according to the United Nations Statistics Division’s M49 standard geoscheme for Eastern Africa. This classification includes the following 20 countries: Burundi, Comoros, Djibouti, Eritrea, Ethiopia, Kenya, Madagascar, Malawi, Mauritius, Mayotte, Mozambique, Réunion, Rwanda, Seychelles, Somalia, South Sudan, Tanzania (United Republic of Tanzania), Uganda, Zambia, and Zimbabwe (United Nations Statistics Division, Standard Country or Area Codes for Statistical Use [M49], available at: https://unstats.un.org/unsd/methodology/m49/). Studies were eligible for inclusion if they were conducted in, or drew their sample population from, one or more of these countries. This definition was applied consistently across all stages of the search, screening, and eligibility assessment. The geographical restriction to East Africa was operationalized in database searches by combining the disease-specific search terms with country names and regional terms corresponding to the above UN M49 classification. Where a study spanned multiple regions, it was included only if data specific to one or more of the 20 Eastern African countries could be identified and extracted independently.

The geographical scope was operationalized at two stages. First, no country filter was applied at the database search stage to maximize sensitivity. Second, during title/abstract and full-text screening, studies were included only if they reported primary data from one or more of the above-listed East African countries. Studies conducted outside this predefined list were excluded. Multi-country studies were included if they provided disaggregated data for at least one eligible East African country.

We note that, as reflected in the results, prevalence studies were ultimately identified in four of these 20 countries (Ethiopia, Kenya, Rwanda, and Uganda), representing 30% of the defined region. The explicit use of the UN M49 classification ensured that the geographical scope was transparently defined, consistently applied, and fully reproducible by independent researchers.

### Grey literature search

To minimize publication bias and ensure comprehensive identification of relevant evidence, a systematic grey literature search was conducted in parallel with the structured electronic database searches. From January 2024 to March 2026, the following sources were searched: Google Scholar, ClinicalTrials.gov, conference abstract repositories of the American Society of Tropical Medicine and Hygiene (ASTMH) and the International Society for Neglected Tropical Diseases (ISNTD), Ministry of Health reports and national Neglected Tropical Disease (NTD) program reports, institutional repositories, and unpublished organizational reports including program evaluations and documents from the Mossy Foot Treatment and Prevention Association (MFTPA) and the END Fund. ClinicalTrials.gov was specifically searched to identify any ongoing or completed intervention studies that may have reported podoconiosis prevalence or health and economic burden data as primary or secondary outcomes, given the potential for such data to remain unpublished in the peer-reviewed literature.

The same search terms applied in the structured database search were used across all grey literature sources: ‘podoconiosis’ OR ‘mossy foot’ OR ‘non-filarial elephantiasis,’ combined with country and regional terms corresponding to the UN M49 Eastern Africa classification. For Google Scholar, where exhaustive retrieval was not feasible, the first 200 results were screened following established practice in systematic reviews [26]. Ministry of Health websites and institutional repositories were searched both systematically, through direct visits to the official websites of all 20 UN M49 Eastern African country health ministries, and opportunistically, through known sources identified via expert contacts with knowledge of national NTD program activities in the region.

All retrieved grey literature records were screened at the abstract level by the same two independent reviewers who conducted the structured database screening, ensuring consistency of the eligibility assessment process across all sources. Disagreements between reviewers were resolved through team discussion and consensus, with the senior author (MS) serving as the designated third reviewer in cases where consensus between the two primary reviewers could not be reached. No language restrictions were applied beyond English and French, reflecting the official and widely used languages of scientific and governmental communication across the Eastern African region. Records meeting the eligibility criteria at abstract screening were sought for full-text retrieval and subjected to the same full-text eligibility assessment applied to records identified through the structured database search, with grey literature sources meeting the inclusion criteria incorporated into the PRISMA flow diagram and included in the review.

As for the database searches, the grey literature search was updated to cover the period January 2024 to March 2026, using the same sources, search terms, screening procedures, and eligibility criteria applied in the original search.

**Fig 2:**
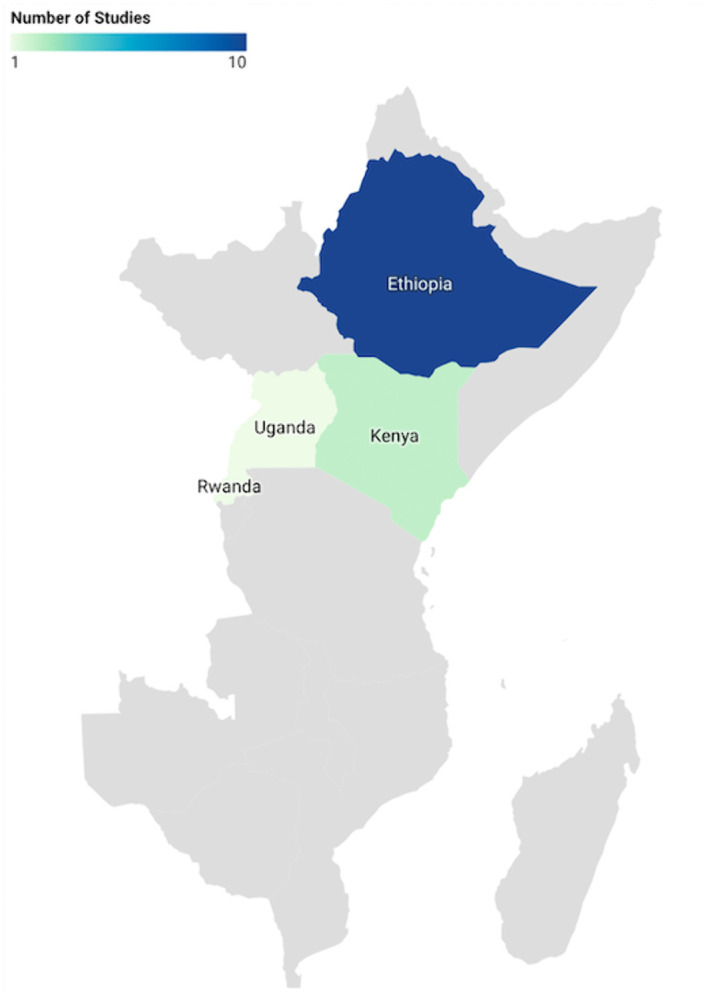
Map of podoconiosis prevalence studies in East Africa.

In addition to the prevalence search, a parallel systematic search was conducted to identify studies reporting the health and economic burden of podoconiosis in Eastern Africa. This search used the same seven electronic databases, geographical restrictions, and date parameters as the prevalence search, with search terms extended to include burden-specific terminology: ‘DALYs,’ ‘disability-adjusted life years,’ ‘economic burden,’ ‘economic impact,’ ‘cost,’ and ‘productivity loss.’ The screening for eligibility for this arm of the review is described in Fig 2 below.

### Study selection

We initially reviewed search results based on their titles and abstracts, followed by a thorough examination of the full texts. During initial screenings, we excluded abstracts that did not describe observational studies, those that did not report the prevalence of podoconiosis in an East African country or case reports on the existence of podoconiosis. We excluded studies that were not based on original data (*i.e., review articles*). Abstracts reporting observational and economic studies focusing on podoconiosis were eligible for full-text review. Articles based on population data were independently assessed for potential inclusion in the review and were considered if they reported the prevalence of podoconiosis or provided sufficient data for prevalence calculation. We excluded health facility-based studies. We also excluded studies that reported the cost of podoconiosis program interventions, those that reported the cost per podoconiosis case and the effectiveness of intervention, and economic studies that only reported the willingness to pay. Lastly, we excluded all studies that were published before 2011, assuming that more recent studies might be more accurate burden estimations and reflect changes in the prevalence of podoconiosis, particularly due to the global attention following the WHO recognition of the disease.

### Study eligibility and quality assessment

Two authors (NH and UUN) screened titles and abstracts of the identified studies to determine their relevance for the review. In case of disagreements, a third author (VA) was involved to resolve any discrepancies. Two authors (NH and UUN) independently retrieved and evaluated the full-text articles. In case of discrepancies, they planned to refer to two other authors (MS and NH) for resolution. One author (NH) evaluated the quality of the studies through use of the Newcastle-Ottawa Scale (NOS) [27]. The NOS allowed an evaluation of each selected study on four criteria: the representativeness of the study sample, the ascertainment of outcome (diagnosis criteria), comparability between different subgroups, and the robustness of the statistical analysis (including the assessment of reporting bias).

### Data extraction and statistical analyses

All articles identified through the searches were exported to Zotero [28]. A data extraction form was developed in Microsoft Excel to record the study author, publication year, study design, East African country, sample size, number of cases, and prevalence data with their corresponding 95% confidence intervals. We created extraction tables using Microsoft Excel and developed data visualizations (maps) using Datawrapper [29,30].

Given the expected variation in prevalence estimates across studies and settings, a random-effects meta-analysis was conducted to pool podoconiosis prevalence data. Prior to pooling, prevalence proportions were stabilized using the Freeman-Tukey Double Arcsine Transformation (FTDAT), which is recommended for meta-analyses of proportions where estimates approach 0 or 1, or where sample sizes vary substantially across studies. This transformation addresses the violation of the normality assumption inherent in untransformed proportions and prevents the confidence intervals of extreme estimates from exceeding the 0–1 boundary. Back-transformation of pooled estimates to the original proportion scale was performed following meta-analysis to aid interpretability.

The random-effects model was specified using the DerSimonian-Laird method-of-moments estimator for the between-study variance (τ²), which is the default estimator implemented in STATA’s metaprop command. Pooled prevalence estimates are presented with 95% confidence intervals. Between-study heterogeneity was quantified using the I² statistic, which describes the percentage of total variation across studies attributable to heterogeneity rather than sampling error, and Cochran’s Q statistic with its corresponding p-value. Heterogeneity was interpreted using conventional thresholds: I² values of 25%, 50%, and 75% were considered to represent low, moderate, and high heterogeneity, respectively. To explore potential sources of heterogeneity, subgroup analyses were conducted and are reported separately. We used funnel plots to present the level of heterogeneity in selected studies.

All statistical analyses were performed using STATA version 18 (StataCorp, College Station, TX, USA).

## Results

The original search from the seven electronic databases identified 774 records. An updated search across the same databases, covering the period January 2024 to March 2026, retrieved a further 12 records, bringing the total to 786 records.

Among the retrieved twelve additional records, two were screened at the title and abstract level, and one proceeded to full-text review. One potentially eligible study was identified: Daniel et al. (2025), a community-based cross-sectional survey conducted in Hawella Tula district, Sidama Region, Ethiopia, between February and May 2024. Following full-text eligibility assessment, this study was excluded on the grounds that its denominator comprised preselected elephantiasis cases identified through the local neglected tropical disease office report rather than a random sample of the general population. As such, the derived prevalence figure is not population-representative and is not directly comparable to the community-based population surveys included in this review. The exclusion was documented in the updated PRISMA flow diagram (Fig 1). No additional studies on DALY estimates or economic burden meeting the inclusion criteria were identified from any other East African country during this period, and no additional studies or reports meeting the inclusion criteria were identified from the updated grey literature search.

Following the removal of 67 duplicates, 719 records were screened at the title and abstract level. Of these, 663 records were excluded at the screening stage: 511 were not podoconiosis studies, 118 were conducted outside the Eastern African region as defined by the United Nations Statistics Division M49 geoscheme, and 34 did not report prevalence data. The remaining 56 records were sought for full-text retrieval. An additional 15 reports identified through manual searching of reference lists were added at this stage, yielding 71 reports assessed for full-text eligibility.

Following full-text review, 57 reports were excluded for the following reasons: 18 were duplicates, 4 were systematic or narrative reviews, 18 were published before the 2011 eligibility cut-off, 3 were case reports, 2 were policy briefs, 1 was excluded due to a non-population-representative denominator (pre-selected elephantiasis cases rather than a random community sample), and 11 were conducted in countries outside the 20-country UN M49 Eastern Africa classification. Ultimately, 14 prevalence studies met all inclusion criteria and were included in the systematic review and meta-analysis.

The included prevalence studies were from four countries in the Eastern African region (Ethiopia, Kenya, Rwanda, and Uganda), equivalent to 15% in the region. Ethiopia had the highest number of studies (n = 10 studies), representing about 71% of the total number of studies included in this review, while the remaining percentage of studies were conducted in Kenya (n = 2 studies), Uganda (n = 1 study), and Rwanda (n = 1 study).

Each country (in Fig 2) is shaded to indicate the presence of included studies, with study locations plotted at the district or regional level where geographic coordinates were available.

The map was created using Datawrapper (https://www.datawrapper.de), an online data visualization tool. The base map layer is sourced from Natural Earth (https://www.naturalearthdata.com), a public domain map dataset freely available for use without restriction. No copyright permission is required for reproduction of the base map layer. Country boundaries reflect the United Nations Statistics Division M49 geoscheme for Eastern Africa as applied in this review.

Data from the four countries (summarized in Table 1) were included in the analyses. Cumulatively, included studies had a total number of 11,782 cases and a total sample size of 1,720,437 (Table 1).

**Table 1:** Characteristics  of included podoconiosis prevalence studies in East Africa (2011–2023).

Study	Country	Year	Urban/Rural Setting	Study Design	Geographic Scope	Location	Key Notes	Prev. % (95% CI)
**Alemu et al.**	**Ethiopia**	2011	**Rural**	**Community-based**	District-level	Gulliso woreda, West Ethiopia (highland)	*Household survey of 26 rural kebeles; subsistence farmers walking barefoot on volcanic red clay*	**2.79%** (2.67–2.92)
**Geshere Oli et al.**	**Ethiopia**	2012	**Rural**	**Health Facility-assisted**	District-level	Midakegn district, central Ethiopia	*House-to-house survey; cases examined at nearby health centre; serological exclusion of LF*	**7.43%** (6.26–8.79)
**Molla et al.**	**Ethiopia**	2012	**Rural**	**Community-based**	Zone-level	East & West Gojam Zones, northern Ethiopia (highland)	*17,553 households across 20 randomly selected kebeles; largest single-zone community survey*	**3.30%** (3.19–3.50)
**Ayele et al.**	**Ethiopia**	2013	**Rural**	**Community-based**	District-level	Bedele Zuria woreda, West Ethiopia	*Community-based survey in Oromia highlands; agricultural communities*	**5.65%** (5.12–6.23)
**Deribe et al.**	**Ethiopia**	2015	**Mixed**	**Nationwide Mapping**	National	Ethiopia-wide; 1,315 communities, 659 woredas, all 9 regional states	*First nationwide integrated podoconiosis & LF mapping; Bayesian multilevel modelling; includes rural & peri-urban*	**4.00%** (3.90–4.10)
**Bekele et al.**	**Ethiopia**	2016	**Rural**	**Community-based**	District-level	Wayu Tuka woreda, East Wollega, West Ethiopia	*Two-phase: census of all district households + clinical assessment phase; subsistence barefoot farmers*	**3.05%** (2.90–3.20)
**Elias et al.**	**Ethiopia**	2016	**Rural**	**Community-based**	District-level	Soddo Zuria district, Wolaita Zone, South Ethiopia	*Rural agricultural community; structured questionnaire + clinical observation; risk factor analysis*	**5.40%** (4.30–6.70)
**Muli et al.**	**Kenya**	2017	**Rural**	**Community-based**	Sub-county level	Mt. Elgon area, highland Kenya	*Small community-based survey in volcanic highland; narrow geographic scope; wide CI due to small N*	**3.40%** (1.80–5.70)
**Kihembo et al.**	**Uganda**	2017	**Rural**	**Community-based**	District-level	Kamwenge district, Western Uganda	*Population-based; focus on risk factor analysis; large N yields tight CI despite low prevalence*	**0.10%** (0.08–0.13)
**Deribe et al.**	**Rwanda**	2019	**Mixed**	**Nationwide Mapping**	National	Rwanda-wide; 80 clusters across all 30 districts	*Largest sample in dataset (N = 1.36M); community health workers conducted census; standardised diagnostic algorithm; FTS and Wb123 LF exclusion*	**0.07%** (0.03–0.08)
**Dejene et al.**	**Ethiopia**	2019	**Rural**	**Community-based**	District-level	Dano district, West Shewa Zone, central Ethiopia	*Community-based; blood sampling for LF exclusion; shoe-wearing age and foot-washing identified as risk factors*	**6.27%** (4.64–8.42)
**Getie et al.**	**Ethiopia**	2020	**Rural**	**Community-based**	Zone-level	Waghmra Zone, Amhara Region, North Ethiopia	*Adult population; multistage sampling; small sample size contributes to wider CI*	**4.30%** (3.00–5.60)
**Sultani et al.**	**Kenya**	2021	**Mixed**	**Nationwide Mapping**	National	Kenya-wide; 48 villages, 24 sub-counties, 15 counties	*First nationwide podoconiosis mapping in Kenya; POC antigen test + physical examination; highland and non-highland areas included*	**0.26%** (0.10–0.50)
**Getachew et al.**	**Ethiopia**	2022	**Rural**	**Community-based**	Zone-level	Gamo Zone, Southern Ethiopia	*Multistage sampling and binary logistic regression for risk factors; soap use and shoe wearing identified as protective*	**6.15%** (4.30–8.00)

**Setting:** Rural = exclusively rural agricultural communities; Mixed = both rural and urban/peri-urban populations included (nationwide surveys).

**Design:** Community-based = household or community-level cross-sectional survey within a defined district or zone; Nationwide Mapping = population-based cross-sectional survey covering multiple districts or the entire country; Health Facility-Assisted = house-to-house identification with clinical confirmation at the nearest health center.

**Abbreviations:** CI = 95% confidence interval; LF = lymphatic filariasis; FTS = Filariasis Test Strip; Wb123 = anti-Wb123 IgG4 serological test; POC = point-of-care.

Stratifying the 14 included studies by sample size threshold (N < 1,000 vs. N ≥ 1,000) revealed a consistent and epidemiologically important pattern: smaller studies uniformly reported higher podoconiosis prevalences with wider confidence intervals, while larger studies captured a broader range of estimates, including the near-zero prevalences from Uganda and Rwanda, with substantially greater precision.

Prevalence estimates varied substantially across studies, ranging from 0.07% (95% CI: 0.03–0.08) in the Rwanda nationwide survey (Deribe et al., 2019) to 7.43% (95% CI: 6.26–8.79) in a district-level study in central Ethiopia (Geshere Oli et al., 2012). This more than 100-fold variation in crude prevalence reflects the profound influence of study design, geographical setting, and target population on observed estimates and is a primary driver of the high between-study heterogeneity observed in the meta-analysis (I² = 99.0%).

Ethiopian studies consistently reported higher prevalence estimates than those from Kenya, Uganda, or Rwanda, with district-level estimates in Ethiopia ranging from 2.79% (Alemu et al., 2011) to 7.43% (Geshere Oli et al., 2012), and most falling between 3% and 6%. The two Kenyan studies reported substantially lower prevalence estimates of 3.40% (Muli et al., 2017) and 0.26% (Sultani et al., 2021), with the latter derived from a large nationwide mapping survey (n = 6,228) likely reflecting population-averaged rather than endemic-area-specific prevalence. The single Ugandan study (Kihembo et al., 2017) reported a prevalence of 0.10% (95% CI: 0.08–0.13) from a very large nationwide sample (n = 51,553). The single Rwandan study (Deribe et al., 2019), which was the largest included study by sample size (n = 1,360,612), reported the lowest prevalence of all included studies at 0.07%.

The variation in sample sizes across studies is also noteworthy and directly influences the weighting of individual studies in the forest plot. Sample sizes ranged from 385 participants (Muli et al., 2017, Kenya) to 1,360,612 participants (Deribe et al., 2019, Rwanda), a difference of more than three orders of magnitude. Studies with larger sample sizes and narrower confidence intervals, such as Deribe et al. (2019), Alemu et al. (2011), and Molla et al. (2012), carried greater weight in the pooled estimate, while smaller district-level studies with wider confidence intervals, contributed proportionally less. It should be noted that the Freeman-Tukey Double Arcsine Transformation applied prior to pooling partially mitigates the instability of extreme prevalence proportions in smaller studies, enabling more stable pooling across this heterogeneous evidence base.

**Fig 3:**
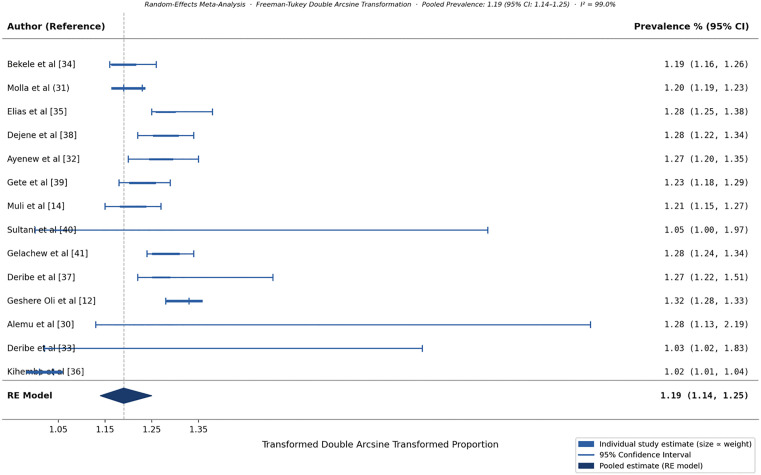
Forest plot: Pooled podoconiosis prevalence.

Fig 3 below presents the forest plot for the pooled podoconiosis prevalence across all 14 included studies.

The random-effects meta-analysis yielded a pooled podoconiosis prevalence of 1.19 (95% CI: 1.14–1.25) on the transformed scale across 14 studies from four Eastern African countries, with substantial between-study heterogeneity (I² = 99.0%).

The pooled prevalence reflects a moderate overall burden of podoconiosis across Eastern Africa, though this estimate should be interpreted cautiously given the high heterogeneity (I² = 99.0%) and the concentration of included studies in Ethiopia. The dominance of Ethiopian data in the evidence base means the pooled estimate is heavily influenced by Ethiopian district-level prevalence figures and may not be generalizable to the broader Eastern African region.

**Fig 4:**
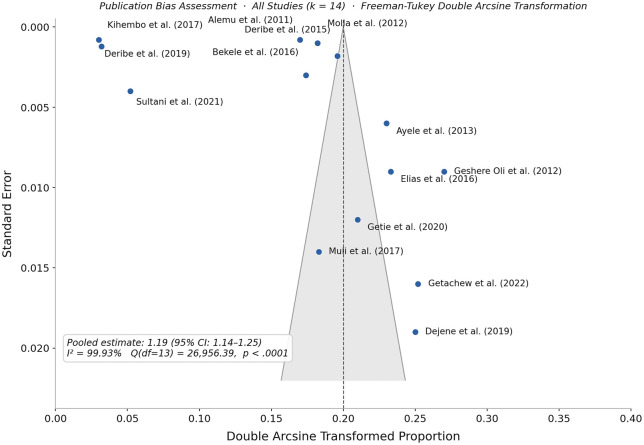
Funnel plot (heterogeneity assessment).

The high I² value (99.3%) as shown in Fig. 4 indicated substantial heterogeneity among the studies even though the random-effects model was applied to account for this. The test for heterogeneity (Q-test) was also highly significant, reinforcing the presence of heterogeneity.

The funnel plot for the full meta-analysis (k = 14 studies) is presented in Fig 4. Visual inspection of the funnel plot revealed a notably asymmetric distribution of studies around the pooled transformed estimate of 1.19 (95% CI: 1.14–1.25), consistent with the presence of substantial heterogeneity across the included studies.

The majority of studies clustered near the apex of the funnel at low standard errors, reflecting the large sample sizes of several included surveys, particularly the Ethiopian nationwide mapping studies (Alemu et al., 2011; Molla et al., 2012; Deribe et al., 2015) and the Rwandan country-wide survey (Deribe et al., 2019). However, the distribution was visibly skewed, with a notable concentration of studies to the right of the pooled estimate in the lower portion of the funnel, including Geshere Oli et al. (2012), Elias et al. (2016), Dejene et al. (2019), and Getachew et al. (2022), suggesting that smaller studies conducted in highly endemic Ethiopian districts reported disproportionately higher prevalence estimates relative to the pooled figure. Three studies fell substantially to the left of the pooled estimate and outside or at the boundary of the 95% confidence interval funnel: Kihembo et al. (2017), reflecting the markedly lower podoconiosis prevalence observed in Uganda where the disease is geographically restricted to highland volcanic areas; Deribe et al. (2019), reflecting Rwanda’s comparatively lower national burden attributable in part to widespread footwear use and national prevention policies; and Sultani et al. (2021), reflecting Kenya’s low population-averaged prevalence as estimated from a large nationwide mapping survey.

The positioning of these three studies outside or at the funnel boundary underscores the substantial epidemiological heterogeneity between countries rather than indicating publication bias per se. The observed funnel plot asymmetry is therefore more plausibly explained by genuine between-study heterogeneity arising from true epidemiological differences across countries and settings, variation in study design and target population, and the concentration of included studies in Ethiopia, than by selective non-publication of studies with low or non-significant prevalence estimates.

This interpretation is consistent with the exceptionally high heterogeneity observed in this meta-analysis (I² = 99.93%; Q(df = 13) = 26,956.39, p <.0001). Given the small number of included studies (k = 14) and this exceptionally high between-study heterogeneity, formal statistical testing of funnel plot asymmetry using Egger’s regression test was considered unreliable, and its results are not reported, consistent with recommendations from the Cochrane Handbook (Higgins et al., 2022). Assessment of publication bias was therefore based on visual inspection of the funnel plot alone. The findings from this assessment should be considered alongside the subgroup analyses presented below, which provide a more detailed examination of the country-level sources of heterogeneity in this review.

Given the dominance of Ethiopian studies in the overall evidence base and the substantial between-country heterogeneity identified through visual inspection of the funnel plot, a subgroup analysis restricted to Ethiopian studies was conducted to obtain a more homogeneous and contextually interpretable prevalence estimate. Ethiopia contributed 10 of the 14 included studies, representing the largest and most geographically diverse body of podoconiosis prevalence data in Eastern Africa, and its district-level estimates consistently exceeded those reported from Kenya, Uganda, and Rwanda.

Restricting the analysis to Ethiopian studies alone allowed for a more precise characterization of the podoconiosis burden within the country with the highest globally recognized disease burden, while minimizing the confounding influence of the marked cross-country epidemiological differences observed in the full meta-analysis. The results of the Ethiopian subgroup analysis, including the corresponding forest plot and funnel plot, are presented in the following sections.

Table 2 presents the characteristics and prevalence estimates of the Ethiopian studies included in this subgroup analysis, comprising all cross-sectional surveys conducted between 2011 and 2023 that met the eligibility criteria and reported population-based podoconiosis prevalence data from Ethiopian districts or regions.

**Table 2:** Characteristics, sample sizes, and prevalence estimates of podoconiosis studies included in the Ethiopian subgroup analysis (2011–2022).

Author (Reference)	Country	Year	Study Design	Cases	Sample Size	Prevalence % (95% CI)
Alemu et al [31]	Ethiopia	2011	Cross-sectional	1,935	69,465	2.79 (2.67–2.92)
Geshere Oli et al [12]	Ethiopia	2012	Cross-sectional	123	1,656	7.43 (6.26–8.79)
Molla et al [32]	Ethiopia	2012	Cross-sectional	1,704	51,017	3.30 (3.19–3.50)
Ayele et al [33]	Ethiopia	2013	Cross-sectional	379	6,710	5.65 (5.12–6.23)
Deribe et al [34]	Ethiopia	2015	Cross-sectional	5,253	129,959	4.00 (3.90–4.10)
Bekele et al [35]	Ethiopia	2016	Cross-sectional	1,197	39,256	3.05 (2.90–3.20)
Elias et al [36]	Ethiopia	2016	Cross-sectional	80	1,483	5.40 (4.30–6.70)
Dejene et al [37]	Ethiopia	2019	Cross-sectional	40	638	6.27 (4.64–8.42)
Getie et al [38]	Ethiopia	2020	Cross-sectional	34	792	4.30 (3.00–5.60)
Getachew et al [39]	Ethiopia	2022	Cross-sectional	42	683	6.15 (4.30–8.00)

The 10 Ethiopian studies included in the subgroup analysis were all community-based cross-sectional surveys conducted between 2011 and 2023, collectively examining 301,659 individuals across multiple districts spanning the major podoconiosis-endemic regions of the country. Prevalence estimates ranged from 2.79% (95% CI: 2.67–2.92; Alemu et al., 2011) to 7.43% (95% CI: 6.26–8.79; Geshere Oli et al., 2012), with larger nationwide surveys consistently reporting lower population-averaged estimates in the 3–4% range, while smaller district-level studies focused on known endemic areas reported higher estimates of 5–7%, reflecting genuine subnational variation in disease burden driven by differences in altitude, soil composition, and barefoot exposure across districts.

**Fig 5:**
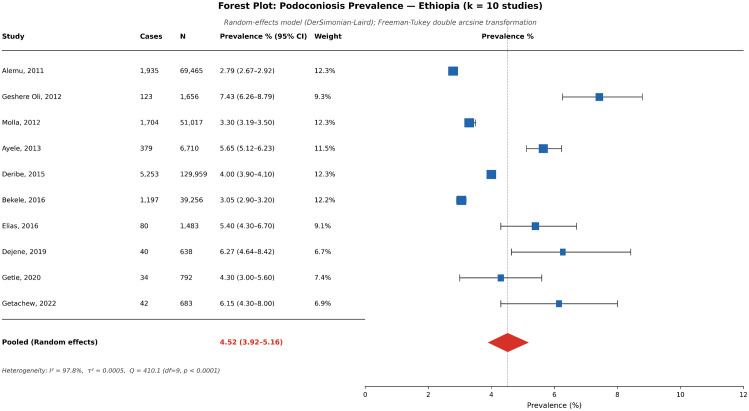
Forest plot: Podoconiosis prevalence—Ethiopia (K = 10 studies).

The temporal distribution of studies across 2011–2023 provides a longitudinal perspective on the Ethiopian podoconiosis burden, though the cross-sectional design of all included studies precludes direct inference about trends in prevalence over time. Notably, the concentration of studies in Ethiopia, representing 10 of the 14 included studies in the full meta-analysis, underscores both the country’s disproportionate share of the regional podoconiosis burden and the relative scarcity of comparable population-based prevalence data from other Eastern African countries, a limitation that should be considered when interpreting the pooled estimates presented in this review. The forest plot for the Ethiopian subgroup (k = 10 studies) is presented in Fig. 5.

The pooled prevalence of podoconiosis across Ethiopian studies was 4.52% (95% CI: 3.92–5.16%), based on a random-effects model using the DerSimonian-Laird estimator with Freeman-Tukey double arcsine transformation. This pooled estimate is substantially higher than that observed in the full 14-study meta-analysis, reflecting the removal of the attenuating effect of the lower-prevalence non-Ethiopian studies from Kenya, Uganda, and Rwanda, and provides a more contextually meaningful estimate of the podoconiosis burden within Ethiopia specifically.

Inspection of the individual study estimates reveals a consistent pattern of higher prevalence in smaller district-level studies compared to larger nationwide surveys. Studies with smaller sample sizes, including Geshere Oli et al. (2012; n = 1,656; prevalence 7.43%), Dejene et al. (2019; n = 638; prevalence 6.27%), and Getachew et al. (2022; n = 683; prevalence 6.15%), reported prevalence estimates substantially above the pooled figure and carried lower weights in the meta-analysis (6.7–9.3%), reflecting their wider confidence intervals and greater uncertainty.

In contrast, large-scale surveys including Alemu et al. (2011; n = 69,465; prevalence 2.79%), Molla et al. (2012; n = 51,017; prevalence 3.30%), and Deribe et al. (2015; n = 129,959; prevalence 4.00%) reported lower prevalence estimates with considerably narrower confidence intervals and carried the highest weights (12.2–12.3%), exerting the greatest influence on the pooled estimate.

This inverse relationship between sample size and observed prevalence is consistent with the hypothesis that smaller studies in this subgroup were predominantly conducted in known highly endemic districts, whereas larger studies captured broader geographic variation, including both endemic and non-endemic areas.

**Fig 6:**
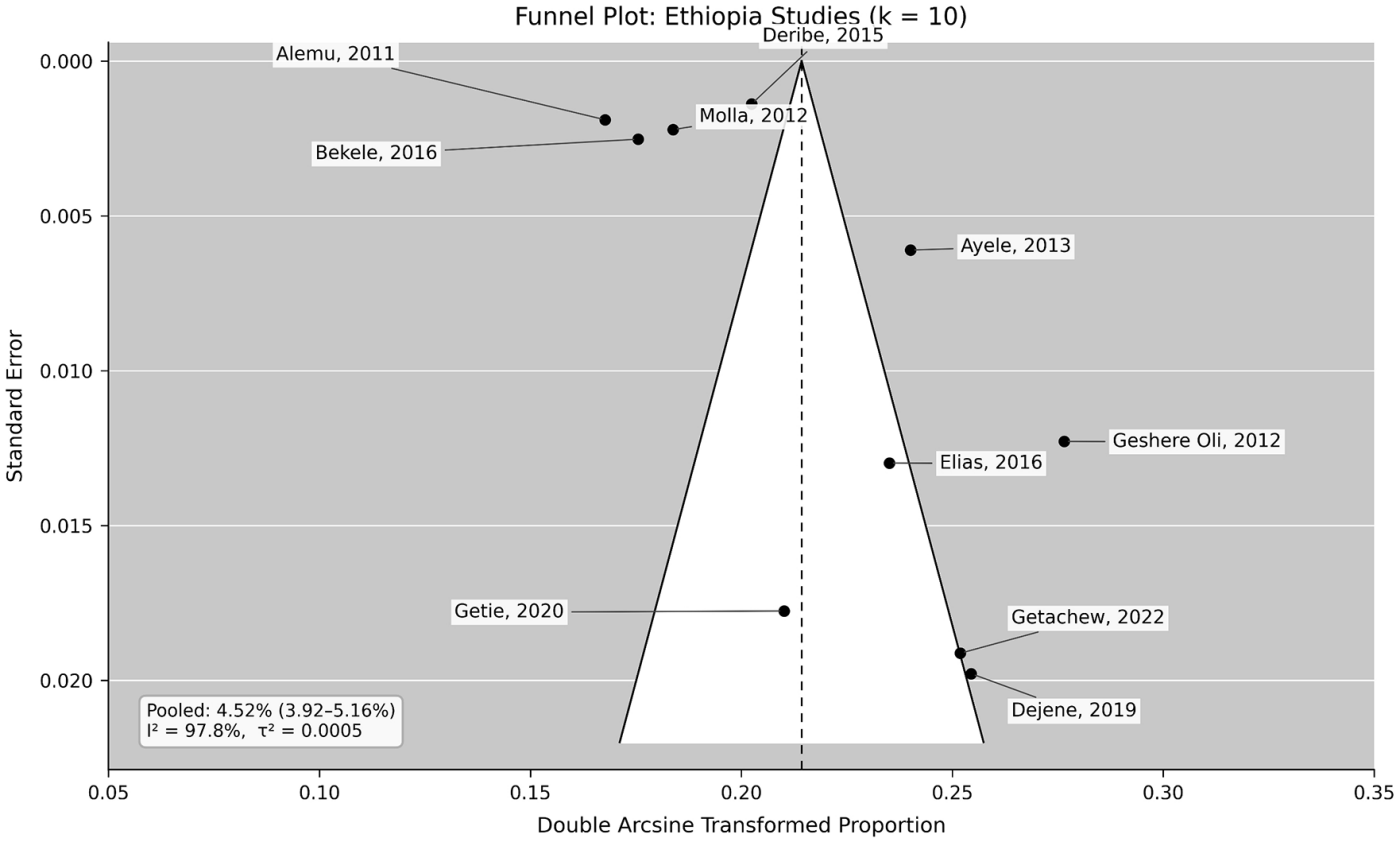
Funnel plot Ethiopia studies (K = 10).

Despite restricting the analysis to Ethiopian studies, the funnel plot revealed persistent asymmetry consistent with substantial between-study heterogeneity (I² = 97.8%; τ² = 0.0005; Q (df = 9) = 410.1, p < 0.0001), driven by genuine subnational variation in podoconiosis burden across Ethiopian districts rather than publication bias. The forest plot for the Ethiopian subgroup (k = 10 studies) is presented in Fig 6.

The residual heterogeneity within Ethiopia likely reflects genuine subnational variation in podoconiosis burden driven by differences in altitude, volcanic soil distribution, barefoot exposure, and access to preventive interventions across districts and regions. These findings underscore the importance of interpreting the pooled prevalence estimate as a summary measure across heterogeneous settings rather than a precise point estimate applicable to any specific Ethiopian district or region.

The funnel plot for the Ethiopia subgroup (k = 10) displays a pattern of asymmetry that, rather than indicating publication bias, is largely consistent with the design-driven small-study effects already identified in the forest plot analysis.

The studies near the apex, Deribe 2015, Molla 2012, Alemu 2011, and Bekele 2016, are the largest and most precise, sitting closest to the top of the plot with the smallest standard errors. Importantly, these large studies cluster to the left of the pooled mean line, reflecting their below-average prevalence estimates, which is precisely what would be expected given that large-scale surveys in this subgroup captured broad geographic variation across both endemic and non-endemic areas, thereby diluting their observed prevalence relative to the pool.

Conversely, the smaller studies—Geshere Oli 2012, Dejene 2019, Getachew 2022, Elias 2016, and Ayele 2013—sit lower on the plot with larger standard errors and are displaced consistently to the right of the pooled mean, reflecting their higher prevalence estimates. This rightward clustering of less precise studies mirrors the forest plot finding that smaller studies in this subgroup were predominantly conducted in known highly endemic districts. Getie 2020 is a minor exception, sitting to the left of center despite its smaller sample size, though this does not meaningfully disrupt the overall pattern.

Overall, the funnel plot asymmetry in this subgroup is best understood as a structural consequence of the heterogeneous study designs contributing to the meta-analysis, specifically, the systematic difference in geographic catchment between small endemic-focused studies and large nationally representative surveys rather than as evidence of selective reporting or publication bias.

The prevalence analyses across this systematic review collectively demonstrate that podoconiosis burden in East Africa is highly geographically concentrated and strongly mediated by study design. The full 14-study pooled estimate provides a broad cross-national reference point, but the subgroup and grouping analyses reveal that this figure obscures more than it communicates. The Ethiopia-specific pooled prevalence of 4.52% (95% CI: 3.92–5.16%) is the most epidemiologically meaningful summary estimate in this review, reflecting the country’s disproportionate contribution to the regional burden and the density of high-quality evidence from its highland endemic zones. Across both the Ethiopia subgroup and the size-based grouping analysis, a consistent inverse relationship emerges between sample size and observed prevalence, driven not by random variation but by the systematic tendency of smaller studies to target confirmed endemic foci while larger surveys capture broader populations that include low-burden areas.

The very high heterogeneity observed throughout, with I² consistently above 95%, should not be considered a statistical inconvenience but rather an epidemiologically meaningful signal, reflecting genuine variation in disease intensity across altitudinal gradients, sampling strategies, and geographic scales.

Any interpretation of pooled estimates from this body of literature must be anchored in an understanding of these design characteristics, and future prevalence research would benefit from standardized sampling frameworks that allow district-level endemic burden to be meaningfully aggregated without the diluting effect of nationwide averages.

While the Ethiopia-specific subgroup analysis highlights the degree to which geographic and altitudinal variation drives heterogeneity within a single country, a parallel pattern emerges when studies are stratified by sample size, as shown in Table 3 below. Grouping studies by whether they enrolled fewer or more than 1,000 participants reveals that design choices — particularly whether a study targeted a known endemic focus or sought national coverage — are as consequential as geography in shaping prevalence estimates. The following section examines these two size-defined groups in turn, drawing on the Ethiopian evidence as a reference point where comparisons are most instructive.

**Table 3:** Study characteristics stratified by sample size: podoconiosis prevalence studies in East Africa (2011–2022).

#	Study	Country	Year	Sample N	Setting	Design	CI Width†	Prevalence % (95% CI)
**Group A — Small Studies (N < 1,000)· 4 studies· All rural· 3 Ethiopia, 1 Kenya· Prevalence range: 3.40–6.27%**								
1	**Dejene et al.**	Ethiopia	2019	638	Rural	Community	±1.89 pp	**6.27%** (4.64–8.42)
2	**Getachew et al.**	Ethiopia	2022	683	Rural	Community	±1.85 pp	**6.15%** (4.30–8.00)
3	**Getie et al.**	Ethiopia	2020	792	Rural	Community	±1.30 pp	**4.30%** (3.00–5.60)
4	**Muli et al.**	Kenya	2017	385	Rural	Community	±1.95 pp	**3.40%** (1.80–5.70)
	**Group Summary**	3 Eth· 1 Ken	2017–2022	385–792	All Rural	All Comm	±1.3–1.9 pp	3.40–6.27%
**Group B — Large Studies (N ≥ 1,000)· 10 studies· 8 Ethiopia, 1 Uganda, 1 Rwanda, 1 Kenya· Prevalence range: 0.07–7.43%**								
1	**Alemu et al.**	Ethiopia	2011	69,465	Rural	Community	±0.13 pp	**2.79%** (2.67–2.92)
2	**Geshere Oli et al.**	Ethiopia	2012	1,656	Rural	HF-assisted	±1.27 pp	**7.43%** (6.26–8.79)
3	**Molla et al.**	Ethiopia	2012	51,017	Rural	Community	±0.16 pp	**3.30%** (3.19–3.50)
4	**Ayele et al.**	Ethiopia	2013	6,710	Rural	Community	±0.56 pp	**5.65%** (5.12–6.23)
5	**Deribe et al.**	Ethiopia	2015	129,959	Mixed	Nationwide	±0.10 pp	**4.00%** (3.90–4.10)
6	**Bekele et al.**	Ethiopia	2016	39,256	Rural	Community	±0.15 pp	**3.05%** (2.90–3.20)
7	**Elias et al.**	Ethiopia	2016	1,483	Rural	Community	±1.20 pp	**5.40%** (4.30–6.70)
8	**Kihembo et al.**	Uganda	2017	51,553	Rural	Community	±0.03 pp	**0.10%** (0.08–0.13)
9	**Deribe et al.**	Rwanda	2019	1,360,612	Mixed	Nationwide	±0.04 pp	**0.07%** (0.03–0.08)
10	**Sultani et al.**	Kenya	2021	6,228	Mixed	Nationwide	±0.20 pp	**0.26%** (0.10–0.50)
	**Group Summary**	8 Eth· 1 Uga· 1 Rwa· 1 Ken	2011–2021	1,483–1,360,612	9 Rural· 3 Mixed	8 Comm· 1 HF· 3 Nat’l	≤±1.3 pp	0.07–7.43%

**†** CI Width = half the width of the 95% confidence interval, expressed in percentage points (pp). Wider values indicate greater sampling imprecision. All 5 small studies reported CI widths ≥ ±1.3 pp; 8 of 9 large studies reported CI widths < ±0.6 pp.

**Abbreviations:** CI = confidence interval; Comm = community-based; Eth = Ethiopia; HF = health facility-assisted; Ken = Kenya; Nat’l = nationwide mapping; Rwa = Rwanda; Uga = Uganda.

**Note:** The N ≥ 1,000 threshold was applied to the total enrolled sample per study. All studies are cross-sectional in design.

### Group A: Small Studies (N < 1,000)

The four studies with fewer than 1,000 participants, all conducted in rural highland communities in Ethiopia (n = 3) and Kenya (n = 1), reported prevalences ranging from 3.40% to 6.27%, with a median of approximately 5.2%. Every study in this group used a community-based cross-sectional design targeting areas already known or suspected to be endemic for podoconiosis. This deliberate sampling in high-burden zones, combined with small geographic catchment areas (single districts or sub-counties), means these estimates reflect local endemic conditions rather than population-level burden. The confidence intervals were correspondingly wide, ranging from ±1.30 to ±1.95 percentage points, indicating substantial sampling uncertainty around each point estimate. These characteristics make small studies informative for describing disease intensity in confirmed endemic foci but unsuitable for generalization to national or regional levels.

### Group B: Large Studies (N ≥ 1,000)

The ten studies with at least 1,000 participants were markedly more heterogeneous in both design and outcome. They spanned all four countries in the review and included both district-level community surveys (n = 7) and nationwide mapping programs (n = 3). Prevalence estimates ranged from 0.07% (Rwanda nationwide, Deribe et al., 2019) to 7.43% (Geshere Oli et al., 2012, Ethiopia), a more than 100-fold difference within a single size stratum. Confidence intervals were considerably narrower than in Group A; eight of ten large studies reported CI widths below ±0.6 percentage points, reflecting the precision gains afforded by larger samples. However, this precision is potentially misleading in the context of the nationwide surveys, where large sample sizes compress confidence intervals around a diluted national average that incorporates both high-burden highland communities and low-burden urban or lowland populations. Notably, the three nationwide surveys (Deribe et al., 2015 Ethiopia; Deribe et al., 2019 Rwanda; Sultani et al., 2021 Kenya) all reported prevalences below 4%, while the seven district- or zone-level Ethiopian studies in this group reported between 2.79% and 7.43%, consistent with the pattern seen in Group A.

Having examined heterogeneity through the lens of sample size and study design, it is informative to consider a complementary geographic stratification, isolating the four studies conducted outside Ethiopia to assess whether the patterns identified in the full analysis and Ethiopian subgroup are specific to Ethiopia or reflect a broader regional phenomenon. This non-Ethiopian subgroup, comprising studies from Kenya, Uganda, and Rwanda, represents a markedly different evidence base in terms of both geographic scale and epidemiological context.

Three of the four studies are large national or near-national surveys, in contrast to the mix of district-level and national designs seen in the Ethiopian subgroup, and none were conducted exclusively in confirmed highland endemic zones with the same deliberateness that characterized many of the Ethiopian studies.

**Fig 7:**
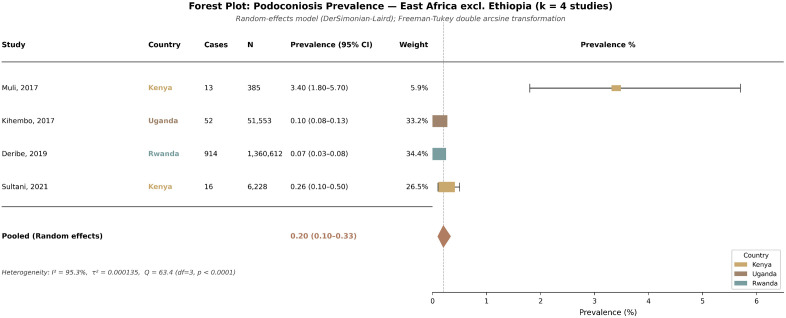
Podoconiosis prevalence—East Africa excluding Ethiopia (K = 4 studies).

The forest plot (Fig 7 below) for this subgroup therefore offers a useful counterpoint to the Ethiopian evidence, not as a directly comparable pooled estimate, but as a means of isolating the contribution of non-Ethiopian settings to the overall heterogeneity and of contextualizing the degree to which the full 14-study pooled prevalence was shaped by Ethiopia’s disproportionate representation in the literature.

The forest plot for the non-Ethiopian subgroup (K = 4 studies) yields a pooled prevalence of 0.20% (95% CI: 0.10–0.33%), which is dramatically lower than both the full 14-study pooled estimate and the Ethiopia-specific pooled prevalence of 4.52%. This stark contrast immediately confirms what the full analysis could only imply, that Ethiopia is the primary driver of the overall pooled estimate, and that the non-Ethiopian evidence base describes a fundamentally different epidemiological picture.

Within this subgroup, the range of individual estimates is itself striking. Muli 2017 (Kenya, n = 385) reports the highest prevalence at 3.40%, though this carries the lowest weight at just 5.9% owing to its very small sample and correspondingly wide confidence interval stretching from 1.80% to 5.70%. At the other extreme, Deribe 2019 (Rwanda, n = 1,360,612) and Kihembo 2017 (Uganda, n = 51,553) report prevalences of 0.07% and 0.10%, respectively, both nationwide or large-scale surveys that dominate the pooled estimate by virtue of their precision, together accounting for over 67% of the total weight. Sultani 2021 (Kenya) sits between these extremes at 0.26%.

Heterogeneity remains very high at I² = 95.3%, which is notable given that only four studies contribute here. This suggests that even within this non-Ethiopian subgroup, the same design-driven variation observed in the full analysis persists; Muli (2017)’s small endemic-focused sampling produces an estimate an order of magnitude higher than the large national surveys. The pooled figure of 0.20% is therefore best understood as heavily anchored by the two largest studies rather than representing a balanced synthesis and should be interpreted alongside the full analysis as evidence that podoconiosis burden outside Ethiopia, while present, is substantially lower and geographically more restricted than within highland Ethiopia.

The prevalence analyses across this systematic review collectively demonstrate that podoconiosis burden in East Africa is highly geographically concentrated, strongly mediated by study design, and disproportionately represented by Ethiopian evidence. The full 14-study pooled estimate provides a broad cross-national reference point, but the cascade of subgroup and grouping analyses undertaken here reveals that this figure obscures more than it communicates. The Ethiopia-specific pooled prevalence of 4.52% (95% CI: 3.92–5.16%) and the non-Ethiopian pooled prevalence of 0.20% (95% CI: 0.10–0.33%) together illustrate the magnitude of the geographic divide within the regional literature, a more than 20-fold difference that the overall pooled estimate can only partially capture.

Across both the Ethiopian subgroup and the size-based grouping analysis, a consistent inverse relationship between sample size and observed prevalence emerges, driven not by random variation but by the systematic tendency of smaller studies to target confirmed endemic foci while larger surveys capture broader populations that incorporate low-burden areas. The non-Ethiopian subgroup reinforces this pattern, with Muli 2017’s small endemic-focused Kenyan estimate standing in stark contrast to the near-zero national prevalences reported from Rwanda and Uganda, suggesting that the design-driven heterogeneity identified in Ethiopia is not uniquely Ethiopian but reflects a structural feature of how podoconiosis has been studied across the region.

The very high heterogeneity observed throughout, with I² consistently above 95% across all analyses, is therefore not a statistical inconvenience but an epidemiologically meaningful signal, reflecting genuine variation in disease intensity across altitudinal gradients, sampling strategies, and geographic scales.

Any interpretation of pooled estimates from this body of literature must be anchored in an understanding of these design characteristics, and future prevalence research would benefit from standardized sampling frameworks that allow district-level endemic burden to be meaningfully aggregated without the diluting effect of nationwide averages.

### DALYs attributed to podoconiosis and associated economic burden

While prevalence estimates provide a foundational understanding of how widely podoconiosis is distributed across East African populations, they capture only part of the disease’s true impact.

Prevalence figures alone cannot convey the lived experience of chronic lymphoedema, the years of productive life lost to disability, or the cascading economic consequences for affected individuals, households, and health systems.

The following sections therefore move beyond epidemiological counts to examine the burden of podoconiosis through two complementary lenses, disability-adjusted life years (DALYs), which quantify the combined toll of premature mortality and lived disability, and direct assessments of economic burden, which translate that toll into the financial costs borne by patients and wider society.

**Fig 8:**
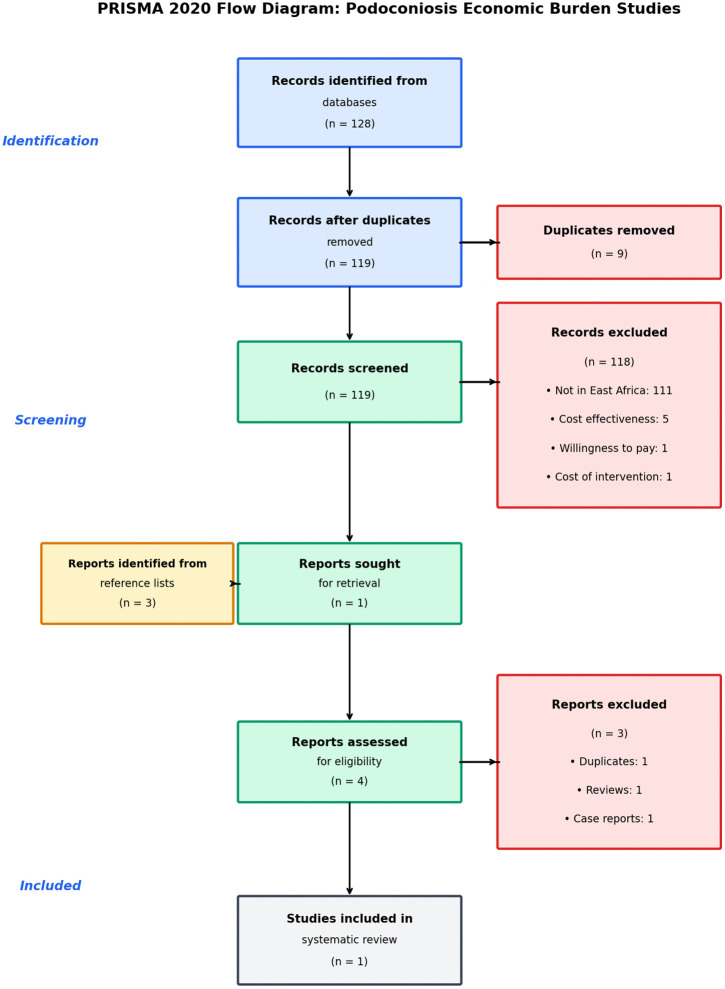
PRISMA flow diagram: Economic burden studies.

Our search generated a total number of sixteen of 128 studies (Fig 8 below) that reported DALYS and economic burden of podoconiosis.

The search identified 128 records relevant to the health and economic burden of podoconiosis in Eastern Africa. Following the removal of 9 duplicates, 119 records were screened at the title and abstract level.

Of these, 118 records were excluded at the screening stage: 111 were conducted outside the Eastern African region as defined by the United Nations Statistics Division M49 geoscheme, 5 reported cost-effectiveness analyses rather than burden estimates, 1 reported willingness-to-pay data, and 1 reported costs of intervention only. The remaining 1 record was sought for full-text retrieval. An additional 3 reports identified through manual searching of reference lists were added at this stage, yielding 4 reports assessed for full-text eligibility.

Following full-text review, 3 reports were excluded: 1 was a duplicate, 1 was a systematic or narrative review, and 1 was a case report. Ultimately, 1 study met all inclusion criteria and was included in the systematic review.

Deribe et al. (2020) reported both the health burden in terms of disability-adjusted life years (DALYs) and the economic burden of podoconiosis. This study, conducted in Ethiopia, estimated that podoconiosis accounted for 172,073 DALYs annually, equivalent to 182 DALYs per 100,000 people based on 1.5 million cases recorded in 2017, with the majority of this burden attributable to chronic lymphoedema rather than acute adenolymphangitis episodes. The total economic burden was estimated at US$213.2 million per year, with productivity losses constituting 91.1% of this figure and an average per-case economic burden of US$136.9.

The absence of comparable DALY or economic burden estimates from Kenya, Uganda, or Rwanda represents a significant gap in the regional literature, precluding any cross-national synthesis of the full disease burden and underscoring the extent to which the quantified burden of podoconiosis in East Africa currently rests on a single national estimate from Ethiopia alone.

## Discussion

The concentration of evidence from Ethiopia is unsurprising given the country’s recognized status as the setting with the highest global burden of podoconiosis. With over 1.5 million estimated cases across 345 endemic districts, Ethiopia has been the primary focus of podoconiosis research for decades, and the volume of available data from this setting naturally dominates any regional synthesis.

The comparatively smaller body of evidence from Uganda is similarly consistent with that country’s lower and more geographically restricted burden, which is predominantly concentrated in the highland volcanic areas of the southwest and east.

Rwanda, by contrast, represents an interesting case: despite its high ecological suitability for podoconiosis, the relatively limited number of studies identified may reflect the impact of the country’s nationwide prevention initiatives, including mandatory footwear policies, which have been among the most structured public health responses to podoconiosis in the region.

Taken together, these country-level patterns suggest that the distribution of research output across East Africa broadly mirrors the burden and policy landscape of each setting, though they also highlight the need for more rigorous longitudinal evidence to assess the effectiveness of prevention programs such as those implemented in Rwanda.

The prevalence analyses in this systematic review demonstrate that podoconiosis burden in East Africa is both substantial and highly heterogeneous, shaped as much by study design as by true geographic variation in disease intensity. The Ethiopia-specific pooled prevalence stands in stark contrast to the non-Ethiopian pooled estimate, a more than 20-fold difference that reflects not only genuine epidemiological variation across the region but also the systematic tendency of smaller endemic-focused studies to report higher prevalence than large national surveys capturing broader populations. The very high heterogeneity observed throughout I² consistently above 95% is an epidemiologically meaningful signal rather than a statistical inconvenience, underscoring the extent to which pooled estimates from this literature must be interpreted with an understanding of the underlying design characteristics of the contributing studies.

Critically, only two countries in the region, Ethiopia and Rwanda, routinely incorporate podoconiosis into their national health information systems (Deribe et al., 2018), meaning that for Kenya and Uganda, the available estimates rest on isolated cross-sectional surveys that cannot capture temporal trends or inform program monitoring in any sustained way.

The burden analyses reveal an equally stark evidence gap. Across all 14 studies retrieved, only one, Deribe et al. (2020), reported both DALY and economic burden estimates, and this was confined to Ethiopia alone. The absence of comparable estimates from Kenya, Uganda, and Rwanda means that the quantified burden of podoconiosis across East Africa currently rests on a single national model, precluding any meaningful cross-national synthesis and rendering the true regional burden effectively unknown.

This gap is not incidental but reflects a systemic pattern of underinvestment in podoconiosis research, compounded by the disease’s continued exclusion from the Global Burden of Disease study, the primary framework through which health conditions compete for international attention and funding prioritization (Davey et al., 2018). Together, the prevalence and burden findings of this review make a compelling case that podoconiosis remains profoundly under-researched relative to its impact and that standardized, nationally representative burden assessments across all endemic East African countries are an important priority for informing both policy and resource allocation.

The prevalence and burden estimates identified in this review must further be contextualized against the scale of the population potentially at risk. Environmental suitability modeling has predicted podoconiosis-conducive conditions in 29 African countries, with East Africa accounting for the greatest concentration of suitable environments across 11 countries (Deribe et al., 2020). As of 2020, an estimated 114.5 million people resided in environmentally suitable areas across the continent, of whom 81.7% were located in East Africa, with Ethiopia, Uganda, Rwanda, and Burundi specifically identified as countries with predicted suitability for podoconiosis occurrence (Deribe et al., 2020).

The magnitude of this at-risk population stands in sharp and troubling contrast to the evidence base described in this review: a region harboring over 90 million people in podoconiosis-suitable environments has generated only 14 prevalence studies and a single national burden estimate over several decades of research. This disparity between the predicted scale of exposure and the depth of the epidemiological evidence base reinforces the conclusion that current estimates substantially underrepresent the true burden of podoconiosis in East Africa and that environmental suitability data should be used to actively guide future surveillance and mapping priorities rather than simply serve as a backdrop to existing prevalence literature.

Studies retrieved in this review spanned over a decade, from 2011 to 2023, a period during which podoconiosis prevention and control programs expanded substantially across Ethiopia and other endemic countries. For instance, Ethiopia’s National NTD Master Plan, shoe distribution programs, and community foot hygiene interventions have been active throughout this period, meaning that more recent studies may report lower prevalence in previously high-burden areas due to genuine reductions in transmission rather than methodological differences. Earlier studies, conducted before large-scale intervention rollout, may, on the other hand, report higher prevalence that is not directly comparable to post-intervention estimates, adding a temporal dimension to the heterogeneity that cannot be formally modeled given the available data.

### Strengths and limitations

#### Strengths.

This systematic review used a transparent methodology, including a comprehensive multi-database search strategy, dual independent screening, and quality assessment using the Newcastle-Ottawa Scale, to minimize the risk of selection bias and enhance the reproducibility of the findings. The application of the Freeman-Tukey double arcsine transformation with a random-effects DerSimonian-Laird model represents a methodologically appropriate approach for pooling prevalence data across studies with widely varying sample sizes and proportions, reducing the distorting influence of extreme estimates on the pooled result. The conduct of multiple pre-specified subgroup analyses by country, sample size, and geographic scope adds analytical depth beyond a single pooled estimate, allowing the sources of heterogeneity to be systematically interrogated rather than merely acknowledged. Finally, this review represents a synthesis of podoconiosis prevalence and burden evidence specific to East Africa to date, providing a regionally focused evidence base that complements prior global systematic reviews and offering a more contextually meaningful platform for informing regional policy and program planning.

#### Limitations.

This review is subject to some limitations that should be considered when interpreting its findings. The very high heterogeneity observed across all analyses with I² values consistently exceeding 95% means that pooled prevalence estimates should be interpreted with caution, as they represent a statistical summary of studies that differ substantially in design, geographic scope, and target populations rather than a precise reflection of any single epidemiological reality. The evidence base is heavily skewed toward Ethiopia, which contributed 71% of included studies, meaning that the findings for Kenya, Uganda, and Rwanda rest on a small number of studies, in some cases a single estimate, limiting the conclusions that can be drawn about podoconiosis burden in these countries. The restriction of the review to French and English-language peer-reviewed publications may have introduced language bias, potentially excluding relevant grey literature, government reports, or studies published in other languages that could have altered the pooled estimates or broadened the geographic coverage of the review. Finally, the near-complete absence of DALY and economic burden data outside Ethiopia means that the review cannot provide a comprehensive assessment of the full health and economic impact of podoconiosis across the East African region, representing both a key limitation of the current review and a priority gap for future research.

## Conclusion and recommendations

This systematic review confirms that podoconiosis represents a significant but profoundly under-quantified public health challenge in East Africa, with disease burden disproportionately concentrated in Ethiopian highland communities and the epidemiological record remaining critically sparse across other endemic countries in the region. The extreme heterogeneity observed throughout reflects genuine variation in disease intensity driven by altitudinal gradients, soil composition, and study design, underscoring that pooled estimates alone cannot adequately capture the burden of a disease so tightly coupled to local ecological and socioeconomic conditions.

To address these gaps, future research should prioritize standardized nationally representative prevalence surveys in under-studied endemic countries, the development of DALY and economic burden models beyond Ethiopia, and the integration of podoconiosis into routine national health information systems. At the global level, sustained advocacy for the inclusion of podoconiosis in the Global Burden of Disease study remains essential; without recognition in the world’s primary burden accounting framework, podoconiosis will continue to be overlooked in the funding and policy prioritization processes that determine whether the millions of people living at risk of this entirely preventable disease will ever receive the attention their burden demands.

## Supporting information

S1 Table Data extraction table — Podoconiosis prevalence studies in East Africa. Full data extraction table for all 14 studies included in the systematic review and meta-analysis, covering podoconiosis prevalence studies published in East Africa between 2011 and 2023.
(XLSX)

S2 Table Complete list of all identified studies — Podoconiosis in East Africa. Comprehensive record of all studies identified through database searches (PubMed/MEDLINE, EMBASE, Scopus, Web of Science, EconLit, WHO AFROLIB, and Google Scholar) and reference list searches conducted between October 2023 and March 2026.
(XLSX)

S3 Table Risk of bias assessment — JBI critical appraisal checklist for prevalence studies. Risk of bias assessment for all 14 studies included in the meta-analysis, conducted using the Joanna Briggs Institute (JBI) Critical Appraisal Checklist for Studies Reporting Prevalence Data (8 criteria).
(XLSX)

S4 Table List of podoconiosis prevalence studies (all community surveys) excluded based on year of publication. (DOCX)

S1 Text Full database search strategies. Complete, reproducible search strategies for all seven electronic databases (PubMed/MEDLINE, EMBASE, Scopus, Web of Science, EconLit, WHO AFROLIB, and Google Scholar) used in this systematic review.
(DOCX)

S1 Checklist PRISMA 2020 checklist. Completed Preferred Reporting Items for Systematic Reviews and Meta-Analyses (PRISMA) 2020 checklist for this review. The PRISMA 2020 checklist is reproduced from [25] Used under the Creative Commons Attribution 4.0 International (CC BY 4.0) licence (https://creativecommons.org/licenses/by/4.0/).
(DOCX)
