# Supplementary material for: The health and economic burden of podoconiosis in East Africa: A systematic review and meta-analysis of health outcomes with narrative synthesis of economic evidence
Source: PLoS Negl Trop Dis. 2026 Jun 17;20(6):e0014427. doi: 10.1371/journal.pntd.0014427 (PMC13340807; doi:10.1371/journal.pntd.0014427)
Supplement: S4 Table — (DOCX) [file pntd.0014427.s004.docx]

**S4 Table: List of podoconiosis prevalence studies (all community surveys) excluded based on year of publication**

| *Author* | *Country* | *Publication Year* | *Prevalence % (95% CI)* |
| --- | --- | --- | --- |
| *Price (28)* | *Ethiopia* | *1974* | *4.09 (3.91–4.28)* |
| *Oomen* et al (29) | *Ethiopia* | *1969* | *2.73 (2.67–2.79)* |
| *Mengistu et al (30)* | *Ethiopia* | *1987* | *5.43 (4.64–6.35)* |
| *Kloos et al (31)* | *Ethiopia* | *1992* | *7.45 (5.30–10.52)* |
| *Frommel et al (32)* | *Ethiopia* | *1993* | *5.06 (4.33–5.90)* |
| *Birrie et al (33)* | *Ethiopia* | *1997* | *3.58 (2.83–4.51)* |
| *Desta et al (34)* | *Ethiopia* | *2003* | *5.46 (5.21–5.71)* |
| *Price (35)* | *Burundi* | *1976* | *0.99 (0.77–1.27)* |
| *Price (35)* | *Rwanda* | *1976* | *0.63 (0.53–0.75)* |
| *Jordan et al (10)* | *Tanzania* | *1956* | *2.51(1.10–3.92)* |
| *Crivelli et al (36)* | *Kenya* | *1986* | *3.87 (3.21–4.66)* |
| *Onapa et al (13)* | *Uganda* | *2001* | *4.52 (3.10–6.54)* |
