# Supplementary material for: The health and economic burden of podoconiosis in East Africa: A systematic review and meta-analysis of health outcomes with narrative synthesis of economic evidence
Source: PLoS Negl Trop Dis. 2026 Jun 17;20(6):e0014427. doi: 10.1371/journal.pntd.0014427 (PMC13340807; doi:10.1371/journal.pntd.0014427)
Supplement: S1 Text — Complete, reproducible search strategies for all seven electronic databases (PubMed/MEDLINE, EMBASE, Scopus, Web of Science, EconLit, WHO AFROLIB, and Google Scholar) used in this systematic review. (DOCX) [file pntd.0014427.s005.docx]

**S1 text**

**Full Database Search Strategies**

**The Health and Economic Burden of Podoconiosis in East Africa:**

**A Systematic Review and Meta-Analysis**

PROSPERO Registration: CRD42023432640

Lead Reviewer & Search Conductor: NH (Nadia Hitimana)

Second Reviewer: UUN (Naillah Umutoni Uwimana)

Original Search: October 2023 – January 2024 | Updated Search: March 2026

**1. Overview of the Search Strategy**

This supplementary document presents the complete, reproducible search strategies used in the systematic review and meta-analysis on the health and economic burden of podoconiosis in East Africa. Searches were conducted across seven electronic databases to identify all studies published from 2011 to March 2026 reporting podoconiosis prevalence, disability-adjusted life years (DALYs), or economic burden from East African countries as defined by the UN M49 geoscheme.

Two parallel searches were conducted: (1) a prevalence search and (2) a health and economic burden search. The same seven databases were used for both, with the burden search extending the core disease terms with additional outcome-specific terminology (DALYs, economic burden, productivity loss). An updated search was conducted in March 2026 using the same strings across all databases.

**1.1 PICOS Framework**

The following PICOS framework guided development of the search strategy and eligibility criteria:

| **PICOS Element** | **Definition** | **Key Search Terms Used** |
| --- | --- | --- |
| P — Population | People residing in East Africa (UN M49: Burundi, Comoros, Djibouti, Eritrea, Ethiopia, Kenya, Madagascar, Malawi, Mauritius, Mayotte, Mozambique, Réunion, Rwanda, Seychelles, Somalia, South Sudan, Tanzania, Uganda, Zambia, Zimbabwe) | podoconiosis, mossy foot, non-filarial elephantiasis, endemic elephantiasis, geochemical elephantiasis |
| I — Index Condition | Confirmed podoconiosis (bilateral asymmetric lower limb lymphoedema in barefoot agricultural communities on volcanic highland soils), with or without LF exclusion | prevalence, epidemiology, health burden, public health, population, economic burden, economic impact, DALYs |
| C — Comparator | Not applicable (observational prevalence and burden studies) | — |
| O — Outcomes | Primary: Prevalence (% with 95% CI) Secondary: DALYs, disability weight, productivity loss, economic cost | — |
| S — Study Design | Community-based cross-sectional surveys, nationwide mapping studies, population-based epidemiological studies, economic burden / DALY estimation studies | — |

**1.2 Eligibility Criteria**

| **Inclusion Criteria** | **Exclusion Criteria** |
| --- | --- |
| **Population & Geography**   - Studies conducted in East Africa (UN M49 — 20-country classification) - Human participants ≥15 years - Community-dwelling or population-based sample   **Study Design**   - Cross-sectional surveys, nationwide mapping studies, population-based epidemiological studies - Economic burden / DALY estimation studies with population-level denominators   **Outcomes**   - Prevalence of podoconiosis with a defined denominator and 95% CI - DALYs, disability weight, productivity loss, or economic costs attributable to podoconiosis   **Publication**   - Published 2011 onwards (following WHO recognition of podoconiosis as an NTD) - English language | - Studies conducted outside East Africa (UN M49) - Studies published before 2011 - Case reports, case series, qualitative studies, editorials, commentaries, conference abstracts without data - Systematic or narrative reviews (used as source of primary studies only) - Studies on lymphatic filariasis, leprosy, or other lymphoedema causes only (not podoconiosis) - Studies with no population-representative denominator (e.g. clinic-based case series) - Cost-effectiveness analyses, willingness-to-pay studies, and intervention cost studies (not population-level burden) - Duplicate publications reporting on the same dataset without additional data |

**1.3 Databases Searched**

Seven electronic databases were searched, as reported in the Methods section of the manuscript:

| **Database** | **Provider** | **Coverage** | **Rationale for Inclusion** |
| --- | --- | --- | --- |
| PubMed / MEDLINE | National Library of Medicine (NLM), USA | Inception – Mar 2026 | Primary clinical/public health database; MeSH controlled vocabulary; core NTD literature |
| Embase | Elsevier | 1974 – Mar 2026 | Biomedical & pharmacological literature; Emtree controlled vocabulary; broader coverage than MEDLINE |
| Scopus | Elsevier | 1996 – Mar 2026 | Multidisciplinary; strong African journal indexing; citation tracking |
| Web of Science | Clarivate Analytics | 1900 – Mar 2026 | Multidisciplinary; citation tracking; SCI-EXPANDED, SSCI, ESCI indexes |
| EconLit | American Economic Association (AEB) | 1969 – Mar 2026 | Economics literature; captures economic burden, productivity loss, and DALY cost studies |
| WHO AFROLIB (WHO African Regional Library) | World Health Organization – AFRO | 1948 – Mar 2026 | Regional grey literature; WHO policy documents; African region health reports and NTD programme outputs |
| Google Scholar | Google LLC | Inception – Mar 2026 | Grey literature; conference abstracts; institutional repositories; captures literature not indexed in formal databases |

**1.4 Search Terms and Boolean Logic**

As stated in the manuscript, the following core search terms were used across all databases:

**Disease terms (Block A):**

'podoconiosis' OR 'mossy foot' OR 'non-filarial elephantiasis'

**Outcome/study type terms (Block B):**

'prevalence' OR 'epidemiology' OR 'health burden' OR 'public health'

OR 'population' OR 'economic burden' OR 'economic impact' OR 'DALYs'

**Combined (Boolean operator AND):**

[Block A] AND [Block B]

For the **burden search**, Block B was extended with additional terms: 'DALYs', 'disability-adjusted life years', 'economic burden', 'economic impact', 'cost', 'productivity loss'.

No geographic filter was applied at the database search stage to maximise sensitivity. Geographic restriction to UN M49 Eastern Africa was applied during title/abstract and full-text screening.

**1.5 Updated Search (January 2024 – March 2026)**

An updated search was conducted on 1 March 2026 across the same seven databases using identical search strings, restricted to articles published from 1 January 2024 onwards. This update retrieved a further 12 records for the prevalence search; none met the eligibility criteria after title/abstract screening.

**1.6 Summary of Search Results**

| **Database** | **Prevalence Search (records)** | **Burden Search (records)** | **Notes** |
| --- | --- | --- | --- |
| PubMed / MEDLINE | 312 + 8 | 62 | Highest yield; MeSH indexing captures podoconiosis efficiently |
| Embase | 198 | 31 + 0 | Strong Emtree indexing; broader biomedical coverage than MEDLINE |
| Scopus | 98 + 2 | 9 + 0 | African journal coverage; citation tracking |
| Web of Science | 103 + 2 | 12 + 0 | Multidisciplinary; SCI-EXPANDED and ESCI indexes |
| EconLit | 30 + 0 | 14 + 0 | Specialist economics database; key for burden/DALY literature |
| WHO AFROLIB | 8 + 0 | 0 + 0 | Grey literature; WHO African region reports; programme documents |
| Google Scholar | 25 + 0 | 0 + 0 | Grey literature; first 200 results screened per established practice |
| Manual reference searching | — | — | 15 additional records from reference lists of full-text articles |
| **TOTAL (original + updated)** | **786 total** | **128 total** | **Records before deduplication; figures from PRISMA flow diagrams (Figures 1 & 8 in manuscript)** |

*Note: Record counts are approximate due to deduplication across databases. Total figures (786 prevalence; 128 burden) correspond to those reported in the PRISMA flow diagrams (Figures 1 and 8 of the manuscript). The combined total across both searches before deduplication was 914 records.*

**2. Full Search Strings by Database**

The following pages present the exact search strings entered into each of the seven databases, using the native syntax and field tags of each platform.

**2.1 PubMed / MEDLINE**

*Database: PubMed / MEDLINE (via NLM — https://pubmed.ncbi.nlm.nih.gov/)*

*Date searched: October 2023 (original); 1 March 2026 (updated)*

*Search period: 2011/01/01 – 2026/03/01*

*Records retrieved: 774 (original search) + 12 (updated search) = 786 total (prevalence + burden combined)*

#1 "podoconiosis"[MeSH Terms]

#2 "podoconiosis"[Title/Abstract]

#3 "non-filarial elephantiasis"[Title/Abstract]

#4 "nonfilarial elephantiasis"[Title/Abstract]

#5 "endemic elephantiasis"[Title/Abstract]

#6 "geochemical elephantiasis"[Title/Abstract]

#7 "mossy foot"[Title/Abstract]

#8 #1 OR #2 OR #3 OR #4 OR #5 OR #6 OR #7

#9 "prevalence"[MeSH Terms] OR "prevalence"[Title/Abstract]

#10 "epidemiology"[Subheading] OR "epidemiology"[Title/Abstract]

#11 "health burden"[Title/Abstract]

#12 "public health"[MeSH Terms] OR "public health"[Title/Abstract]

#13 "population"[Title/Abstract]

#14 "economic burden"[Title/Abstract]

#15 "economic impact"[Title/Abstract]

#16 "DALYs"[Title/Abstract] OR "disability-adjusted life years"[Title/Abstract]

#17 "disability-adjusted life year"[MeSH Terms]

#18 "cost*"[Title/Abstract]

#19 "productivity loss"[Title/Abstract]

#20 #9 OR #10 OR #11 OR #12 OR #13 OR #14 OR #15 OR #16 OR #17 OR #18 OR #19

#21 #8 AND #20

*Filters: Publication date: 2011/01/01–present; Language: English*

**2.2 Embase**

*Database: Embase (via Elsevier — https://www.embase.com/)*

*Date searched: October 2023 (original); 1 March 2026 (updated)*

*Search period: 2011–2026*

#1 'podoconiosis'/exp

#2 'podoconiosis':ab,ti

#3 'non-filarial elephantiasis':ab,ti

#4 'nonfilarial elephantiasis':ab,ti

#5 'endemic elephantiasis':ab,ti

#6 'geochemical elephantiasis':ab,ti

#7 'mossy foot':ab,ti

#8 #1 OR #2 OR #3 OR #4 OR #5 OR #6 OR #7

#9 'prevalence'/exp OR 'prevalence':ab,ti

#10 'epidemiology':ab,ti OR 'disease burden':ab,ti

#11 'health burden':ab,ti OR 'public health':ab,ti

#12 'population':ab,ti

#13 'economic burden':ab,ti OR 'economic impact':ab,ti

#14 'disability adjusted life year':ab,ti OR 'DALY':ab,ti

#15 'cost*':ab,ti OR 'productivity loss':ab,ti

#16 #9 OR #10 OR #11 OR #12 OR #13 OR #14 OR #15

#17 #8 AND #16

*Filters: [py]/2011:2026; [lang]/eng*

**2.3 Scopus**

*Database: Scopus (via Elsevier — https://www.scopus.com/)*

*Date searched: October 2023 (original); March 2026 (updated)*

*Search period: 2011–2026*

TITLE-ABS-KEY ( podoconiosis OR "non-filarial elephantiasis"

OR "nonfilarial elephantiasis" OR "endemic elephantiasis"

OR "geochemical elephantiasis" OR "mossy foot" )

AND

TITLE-ABS-KEY ( prevalence OR epidemiology OR "health burden"

OR "public health" OR population OR "economic burden"

OR "economic impact" OR DALYs OR "disability-adjusted life years"

OR cost* OR "productivity loss" )

AND PUBYEAR > 2011

AND LANGUAGE ( english )

AND DOCTYPE ( ar OR re )

**2.4 Web of Science**

*Database: Web of Science Core Collection (Clarivate Analytics)*

*Date searched: October 2023 (original); March 2026 (updated)*

*Indexes searched: SCI-EXPANDED, SSCI, A&HCI, ESCI*

*Search period: 2011–2026*

TS = ( podoconiosis OR "non-filarial elephantiasis"

OR "nonfilarial elephantiasis" OR "endemic elephantiasis"

OR "geochemical elephantiasis" OR "mossy foot" )

AND

TS = ( prevalence OR epidemiology OR "health burden"

OR "public health" OR population OR "economic burden"

OR "economic impact" OR DALY* OR "disability-adjusted life year*"

OR cost* OR "productivity loss" )

*Refined by: Publication Years: 2011–2026; Language: English*

**2.5 EconLit**

*Database: EconLit (American Economic Association, via EBSCOhost)*

*Date searched: October 2023 (original); March 2026 (updated)*

*Search period: 2011–2026*

*Rationale: EconLit was included specifically to capture economic burden studies, DALY cost analyses, and productivity loss literature not indexed in standard biomedical databases.*

TI ( podoconiosis OR "non-filarial elephantiasis" OR "mossy foot" )

OR

AB ( podoconiosis OR "non-filarial elephantiasis" OR "mossy foot" )

AND

AB ( "economic burden" OR "economic impact" OR "disability-adjusted life year*"

OR DALY* OR "productivity loss" OR cost* OR "cost-effectiveness"

OR prevalence OR "public health" OR "health burden" )

*Limiters: Publication date: 2011–2026; Language: English*

*Note: EconLit returned a relatively small number of records as podoconiosis is not well-indexed in economics literature. All retrieved records were screened.*

**2.6 WHO AFROLIB (WHO African Regional Library)**

*Database: WHO AFROLIB — WHO African Regional Library (via WHO AFRO Regional Office)*

*URL: https://afrolib.afro.who.int/*

*Date searched: October 2023/ March 2026 (updated)*

*Rationale: WHO AFROLIB was searched to capture grey literature, regional policy documents, WHO technical reports, Ministry of Health publications, and NTD programme documents from the African region not indexed in formal academic databases.*

Free-text search terms used (entered in search box):

podoconiosis

"non-filarial elephantiasis"

"mossy foot"

podoconiosis AND prevalence

podoconiosis AND burden

podoconiosis AND Ethiopia

podoconiosis AND Kenya

podoconiosis AND Rwanda

podoconiosis AND Uganda

podoconiosis AND "East Africa"

*Note: WHO AFROLIB does not support formal Boolean query syntax. Searches were conducted as free-text queries and results were screened by title. The database was searched to ensure capture of WHO technical reports, guidance documents, and African regional NTD programme publications.*

**2.7 Google Scholar**

*Database: Google Scholar (https://scholar.google.com/)*

*Date searched: October 2023 (original); March 2026 (updated)*

*Rationale: Google Scholar was searched as a supplementary source to capture grey literature, conference proceedings, institutional repositories, and documents from organisations (MFTPA, END Fund, ClinicalTrials.gov) not indexed in formal databases.*

*Search approach: Per established systematic review practice (Haddaway et al., 2015, PLOS ONE), the first 200 search results were screened for each query.*

Search queries entered:

1. podoconiosis prevalence East Africa

2. podoconiosis Ethiopia Kenya Rwanda Uganda prevalence

3. "non-filarial elephantiasis" East Africa prevalence

4. "mossy foot" prevalence burden

5. podoconiosis DALYs economic burden East Africa

6. podoconiosis "health burden" OR "economic impact" Africa

7. podoconiosis filetype:pdf site:who.int OR site:afro.who.int

8. podoconiosis "Ministry of Health" Ethiopia OR Kenya OR Rwanda OR Uganda

*Grey literature sources also searched via Google Scholar:*

- ClinicalTrials.gov: search term "podoconiosis"

- ASTMH conference repository: search "podoconiosis"

- ISNTD conference repository: search "podoconiosis"

- MFTPA (Mossy Foot Treatment and Prevention Association) documents

- END Fund programme evaluations and reports

- Ministry of Health websites: all 20 UN M49 Eastern African countries

*Note: The same search terms were used: 'podoconiosis' OR 'mossy foot' OR 'non-filarial elephantiasis', combined with country and regional terms corresponding to the UN M49 Eastern Africa classification.*

**3. Deduplication and Record Management**

All records retrieved from database searches were exported to Zotero (version 6) for initial reference management. Records were then imported into Covidence (Veritas Health Innovation, Melbourne, Australia) for systematic review management. Deduplication was performed as follows:

1. Automatic deduplication within Zotero using title, author, year, and journal fields
2. Secondary deduplication within Covidence at import using the platform's built-in algorithm
3. Manual verification of borderline cases (same study, different publication formats or preprint/journal pairs)

Prevalence search: 786 records identified → 67 duplicates removed → 719 records screened at title/abstract.

Burden search: 128 records identified → 9 duplicates removed → 119 records screened at title/abstract.

Combined (both searches): 914 records identified → 76 duplicates removed → 838 records screened at title/abstract level.

**4. Screening Process**

**4.1 Title and Abstract Screening**

Title and abstract screening was conducted independently by two reviewers (NH and UUN) using Covidence. In cases of disagreement, a third author (VA) was consulted for resolution. Decision rules applied:

- Include: any study appearing to report podoconiosis prevalence or burden data in East Africa, published 2011 onwards
- Exclude: studies clearly not about podoconiosis (e.g. lymphatic filariasis or leprosy only)
- Exclude: studies clearly conducted outside East Africa (UN M49 classification)
- Exclude: studies published before 2011
- Uncertain: forward to full-text review

**4.2 Full-Text Eligibility Assessment**

Full texts were retrieved and assessed against all eligibility criteria. NH assessed all retrieved full texts, with UUN independently reviewing 20% as a quality check. Reasons for exclusion at full-text stage were recorded and are reported in the PRISMA flow diagrams (Figures 1 and 8 in the manuscript) and in S2 Table.

**4.3 Manual Reference List Searching**

Reference lists of all 71 full-text articles assessed for eligibility (prevalence search) were manually screened by NH. This identified 15 additional potentially eligible records; none met all eligibility criteria after full-text review.

**4.4 Grey Literature**

Grey literature was searched via Google Scholar and WHO AFROLIB (see Section 2.6–2.7). Additional sources searched included: ClinicalTrials.gov, ASTMH and ISNTD conference repositories, Ministry of Health websites for all 20 UN M49 Eastern African countries, and documents from the Mossy Foot Treatment and Prevention Association (MFTPA) and the END Fund. The grey literature search was updated to cover January 2024 – March 2026 using identical sources and search terms.

**5. Reporting Standards**

This systematic review was conducted and reported in accordance with the Preferred Reporting Items for Systematic Reviews and Meta-Analyses (PRISMA) 2020 guidelines.

*Page MJ, McKenzie JE, Bossuyt PM, Boutron I, Hoffmann TC, Mulrow CD, et al. The PRISMA 2020 statement: an updated guideline for reporting systematic reviews.* **BMJ**. 2021;372:n71. doi:10.1136/bmj.n71

*Haddaway NR, Collins AM, Coughlin D, Kirk S. The Role of Google Scholar in Evidence Reviews and Its Applicability to Grey Literature Searching.* **PLOS ONE**. 2015;10(9):e0138237. doi:10.1371/journal.pone.0138237
