# Supplementary material for: The health and economic burden of podoconiosis in East Africa: A systematic review and meta-analysis of health outcomes with narrative synthesis of economic evidence
Source: PLoS Negl Trop Dis. 2026 Jun 17;20(6):e0014427. doi: 10.1371/journal.pntd.0014427 (PMC13340807; doi:10.1371/journal.pntd.0014427)
Supplement: S1 Checklist — Completed Preferred Reporting Items for Systematic Reviews and Meta-Analyses (PRISMA) 2020 checklist for this review. The PRISMA 2020 checklist is reproduced from [25] Used under the Creative Commons Attribution 4.0 International (CC BY 4.0) licence (https://creativecommons.org/licenses/by/4.0/). (DOCX) [file pntd.0014427.s006.docx]

**PRISMA 2020 Checklist**

*The Health and Economic Burden of Podoconiosis in East Africa: A Systematic Review and Meta-Analysis of Health Outcomes with Narrative Synthesis of Economic Evidence*

| **Item** | **Page** | **Checklist item** | **Location / Reported** |
| --- | --- | --- | --- |
| **TITLE** | | | |
| **1** | **p.1** | Identify the report as a systematic review. | **Title:** “The Health and Economic Burden of Podoconiosis in East Africa: A Systematic Review and Meta-Analysis of Health Outcomes with Narrative Synthesis of Economic Evidence”. |
| **ABSTRACT** | | | |
| **2** | **pp.1–3** | See the PRISMA 2020 for Abstracts checklist. | Structured abstract (Background, Methods, Results, Conclusion); PROSPERO number (p.3). |
| **INTRODUCTION** | | | |
| **3** | **pp.5–8** | Describe the rationale for the review in the context of existing knowledge. | Introduction: rationale; GBD absence; East Africa evidence gap. |
| **4** | **p.8** | Provide an explicit statement of the objective(s) the review addresses. | Objectives section (p.8). |
| **METHODS** | | | |
| **5** | **pp.9–11** | Specify inclusion/exclusion criteria and how studies were grouped for the syntheses. | Methods: Study selection (p.11); Geographical scope / UN M49 (p.9); 2011 cut-off. |
| **6** | **pp.8–11** | Specify all databases, registers, websites, organisations, reference lists and other sources searched. | Methods: Database Searches (p.8); Grey Literature Search (pp.10–11); reference-list searching. |
| **7** | **p.8; S4** | Present the full search strategies for all databases, registers and websites, including filters and limits. | Methods: Database Searches (p.8); full strategies in S4 Table (p.49). |
| **8** | **p.11** | Specify methods to decide whether a study met inclusion criteria, number of reviewers, independence, and automation tools. | Methods: Study eligibility and quality assessment (p.11); two independent reviewers (NH, UUN); third reviewer (VA); Zotero. |
| **9** | **p.11** | Specify methods used to collect data from each report, number of reviewers, independence, processes for confirming data. | Methods: Data Extraction and Statistical Analyses (p.11); Excel extraction form; variables listed. |
| **10a** | **pp.8,11** | List and define all outcomes for which data were sought. | Objectives (p.8); Data Extraction (p.11) — prevalence, DALYs, economic burden. |
| **10b** | **p.11** | List and define all other variables for which data were sought. | Data Extraction (p.11): author, year, design, country, sample size, cases, 95% CIs. |
| **11** | **p.11; S3** | Specify methods to assess risk of bias, tool(s) used, and number of reviewers. | Methods: Newcastle-Ottawa Scale (p.11); assessment in S3 Table. |
| **12** | **pp.11–12** | Specify for each outcome the effect measure(s) used in synthesis or presentation. | Statistical Analyses (pp.11–12) — prevalence proportion with 95% CI. |
| **13a** | **pp.11–12** | Describe processes used to decide which studies were eligible for each synthesis. | Study selection (p.11); Statistical Analyses (pp.11–12). |
| **13b** | **p.11** | Describe methods to prepare data for synthesis (missing statistics, data conversions). | Statistical Analyses (p.11) — Freeman-Tukey double arcsine transformation; back-transformation. |
| **13c** | **pp.12,17** | Describe methods to tabulate or visually display results of individual studies and syntheses. | Statistical Analyses (p.12); forest/funnel plots; Datawrapper mapping (p.17). |
| **13d** | **p.12** | Describe methods used to synthesise results and rationale; methods to assess heterogeneity. | Statistical Analyses (p.12): random-effects DerSimonian-Laird; I² and Cochran’s Q. |
| **13e** | **p.12** | Describe methods to explore possible causes of heterogeneity (e.g. subgroup analysis). | Statistical Analyses (p.12): subgroup analyses by country, sample size, geographic scope. |
| **13f** | **pp.24–33** | Describe any sensitivity analyses conducted to assess robustness. | Results: Ethiopian subgroup (pp.24–29); sample-size subgroups (pp.30–33). |
| **14** | **pp.12,22** | Describe methods to assess risk of bias due to missing results (reporting biases). | Statistical Analyses (p.12): funnel plots; funnel-plot inspection (pp.22–23). |
| **15** | **p.11** | Describe methods used to assess certainty in the body of evidence. | Methods NOS quality appraisal (p.11); certainty discussed via heterogeneity. |
| **RESULTS** | | | |
| **16a** | **pp.13–14** | Describe results of the search and selection process, ideally with a flow diagram. | Results (pp.13–14): record counts; Figure 1 (PRISMA 2020 flow, prevalence); Figure 8 economic (p.35). |
| **16b** | **p.13; S2** | Cite studies that might appear to meet criteria but were excluded, and explain why. | Results: exclusion (p.13); full exclusion list in S2 Table. |
| **17** | **pp.17–18** | Cite each included study and present its characteristics. | Results: study characteristics; Table 1/Table 2 (pp.17–18); S1 Table. |
| **18** | **S3** | Present risk-of-bias assessments for each included study. | S3 Table: Newcastle-Ottawa Scale assessment for all 14 studies. |
| **19** | **pp.17–18** | Present summary statistics and effect estimates with CIs for each study and outcome. | Results: prevalence with 95% CIs; forest plots; S1 Table. |
| **20a** | **pp.19–21** | For each synthesis, summarise characteristics and risk of bias among contributing studies. | Results: syntheses described (pp.19–21). |
| **20b** | **pp.15,24** | Present results of all statistical syntheses with summary estimates, CIs and heterogeneity. | Results: pooled 1.19 (95% CI 1.14–1.25) (p.15); Ethiopia 4.52% (p.24); I² = 99.0%. |
| **20c** | **pp.19,24–33** | Present results of all investigations of possible causes of heterogeneity. | Results: subgroup analyses by country (pp.24–29) and sample size (pp.30–33). |
| **20d** | **pp.24–33** | Present results of all sensitivity analyses. | Results: Ethiopian and sample-size subgroup analyses. |
| **21** | **pp.22–23** | Present risk of bias due to missing results for each synthesis. | Results: Funnel plot inspection and interpretation (pp.22–23). |
| **22** | **pp.11,39–40** | Present assessments of certainty in the body of evidence for each outcome. | NOS appraisal (p.11); certainty/limitations discussed (pp.39–40). |
| **DISCUSSION** | | | |
| **23a** | **pp.38–39** | Provide a general interpretation of results in the context of other evidence. | Discussion (pp.38–39). |
| **23b** | **pp.39–40** | Discuss limitations of the evidence included in the review. | Discussion / Limitations (pp.39–40): heterogeneity; Ethiopian dominance; DALY/economic data scarcity. |
| **23c** | **p.40** | Discuss limitations of the review processes used. | Limitations (p.40): single-reviewer quality assessment; English/French restriction. |
| **23d** | **pp.40–41** | Discuss implications for practice, policy, and future research. | Discussion / Conclusion (pp.40–41): standardised surveys; burden modelling beyond Ethiopia; GBD inclusion. |
| **OTHER INFORMATION** | | | |
| **24a** | **p.3** | Provide registration information including register name and number, or state not registered. | Abstract (p.3): PROSPERO CRD42023432640. |
| **24b** | **p.3** | Indicate where the protocol can be accessed, or state none prepared. | PROSPERO record CRD42023432640 (p.3). |
| **24c** | **pp.8,13** | Describe and explain any amendments to registration or protocol information. | Methods (p.8) and Results (p.13): updated March 2026 search described. |
| **25** | **pp.41–42** | Declare sources of financial/non-financial support and the role of funders. | Funding statement (pp.41–42): NIHR (131996), UK Government international development funding. |
| **26** | **pp.44** | Declare any competing interests of review authors. | No competing-interests declaration located: a statement must be added (see note below). |
| **27** | **p.42; S1–S4** | Report which materials are publicly available and where (data, forms, code, other). | Data Availability Statement (p.42): S1–S4 Tables (extraction, study list, risk of bias, search strategies). |

**Attribution**

*From: Page MJ, McKenzie JE, Bossuyt PM, Boutron I, Hoffmann TC, Mulrow CD, et al. The PRISMA 2020 statement: an updated guideline for reporting systematic reviews. BMJ. 2021;372:n71. doi:10.1136/bmj.n71. Used under the Creative Commons Attribution 4.0 International (CC BY 4.0) licence (https://creativecommons.org/licenses/by/4.0/).*
