# Supplementary material for: The health and economic burden of podoconiosis in East Africa: A systematic review and meta-analysis of health outcomes with narrative synthesis of economic evidence
Source: PLoS Negl Trop Dis. 2026 Jun 17;20(6):e0014427. doi: 10.1371/journal.pntd.0014427 (PMC13340807; doi:10.1371/journal.pntd.0014427)
Supplement: Attachment — Submitted filename: Reviewers comments.docx [file pntd.0014427.s007.docx]

**Major gaps Identified**

- ***High Heterogeneity:*** The meta-analysis reports an extremely high $I^2$ value of 99.9%, indicating significant variability between studies that makes the pooled prevalence estimate difficult to generalize.
- ***Geographic Bias:*** Ethiopia dominates the dataset, accounting for 65% of the included studies, which limits the understanding of the burden in neighboring countries like Kenya, Tanzania, and Burundi.
- ***Scarcity of Metrics:*** Only one study in the entire region provided usable DALY and economic cost data, highlighting a severe lack of comprehensive health-economic research outside of Ethiopia.

Failure to use standardized diagnostic criteria led to high levels of methodological heterogeneity.

What will be your justification for this?

All supplementary files need proper formatting in table (Please ensure uniformity of the fonts and writing styles)

| Review Question | Status |  |
| --- | --- | --- |
| Objectives & Hypothesis clearly articulated? | YES |  |
| Study design appropriate? | YES |  |
| Population clearly described? | YES |  |
| Sample size sufficient for power? | **PARTIAL** | Prevalence: Highly sufficient (1.65 million individuals pooled). Economic/DALYs: Insufficient; only one eligible study provided these metrics, which the authors identify as a major regional data gap. |
| Correct statistical analysis used? | YES | Utilized STATA Version 18, a random-effects model for pooled prevalence, and rigorous assessments for heterogeneity ($I^2$) and publication bias (Funnel plots). |
| Ethical/Regulatory requirements met? | YES | No concerns. The study uses secondary data from published literature. Quality and risk of bias were formally assessed using the Newcastle-Ottawa Scale (NOS). |
