# Supplementary material for: The health and economic burden of podoconiosis in East Africa: A systematic review and meta-analysis of health outcomes with narrative synthesis of economic evidence
Source: PLoS Negl Trop Dis. 2026 Jun 17;20(6):e0014427. doi: 10.1371/journal.pntd.0014427 (PMC13340807; doi:10.1371/journal.pntd.0014427)
Supplement: Attachment — Submitted filename: Response_to_PLOS_Reviewers_Comments_SR_April112026.docx [file pntd.0014427.s009.docx]

**Point-by-Point Response to Reviewer Comments**

*Manuscript: The Health and Economic Burden of Podoconiosis in East Africa: A Systematic Review and Meta-Analysis*

*Authors: Nadia Hitimana, Vasso Anagnostopoulou, Stephen Bremner, Naillah Umutoni Uwimana, Natalia Hounsome, Lawrence Rugema, Leon Mutesa, Maya Semrau*

*Date: April 2026*

We thank the Editor and Reviewers for their careful reading of our manuscript and their constructive feedback. We have revised the manuscript thoroughly in response to all comments. Below we provide a point-by-point response to each comment, indicating where changes have been made in the revised manuscript with reference to page and line numbers.

All line numbers refer to the revised manuscript with continuous line numbering(Systematic_Review_FullManuscript_Revised_With_Trackhanges_April112026).

Deleted text from the original submission is shown with strikethrough in the tracked-changes version; new or revised text is shown in the accepted text of the revised manuscript.

**EDITORIAL REQUIREMENTS**

**Comment 1: CRediT Author Contributions**

| **Editor's Comment** | *Please ensure that the CRediT author contributions listed for every co-author are completed accurately and in full. At this stage, the following authors require contributions: Nadia Hitimana, Vasso Anagnostopoulou, Stephen Bremner, Naillah Umutoni Uwimana, Natalia Hounsome, Lawrence Rugema, Leon Mutesa, and Maya Semrau.* |
| --- | --- |
| **Response** | We thank the Editor for this requirement. The Author Contributions section has been revised to include CRediT-compliant contributions for all co-authors. The revised statement (located in the Author Contributions section, lines 1–5 of that section) now reads as follows:  NH: Conceptualization, Methodology, Investigation, Formal Analysis, Writing – Original Draft, Project Administration. VA: Methodology, Formal Analysis, Writing – Review & Editing. SB: Methodology, Formal Analysis, Writing – Review & Editing. UUN: Investigation (data extraction), Writing – Review & Editing. NHs: Conceptualization, Writing – Review & Editing, Supervision. LR: Writing – Review & Editing, Resources. LM: Formal Analysis, Writing – Review & Editing. MS: Conceptualization, Methodology, Writing – Review & Editing, Supervision, Funding Acquisition.  Manuscript location: Author Contributions section (end of main text). |

**Comment 2: Figure File Formats**

| **Editor's Comment** | *Please upload all main figures as separate Figure files in .tif or .eps format.* |
| --- | --- |
| **Response** | Acknowledged. All main figures (Figures 1–8) have been prepared as separate files in .tif format at a minimum resolution of 300 dpi and will be uploaded as individual figure files accompanying this submission. This is a file-submission requirement and no changes to the manuscript text have been made. |

**Comment 3: Supporting Information Legends**

| **Editor's Comment** | *Please add a full list of legends for your Supporting Information files after the references list.* |
| --- | --- |
| **Response** | Acknowledged. A Supporting Information section listing legends for all supplementary files (S1 Table: Data extraction table; S2 Table: Full list of identified studies with exclusion reasons; S3 Table: Risk of bias assessments; S4 Table: Full database search strategies) has been added to the manuscript after the References list. This is a formatting requirement; the relevant content has been added at the end of the manuscript. |

**Comment 4: Figure 2 Copyright**

| **Editor's Comment** | *Figure 2: Please (a) provide a direct link to the base layer of the map and ensure this is also included in the figure legend; and (b) provide a link to the terms of use/license information for the base layer image or shapefile.* |
| --- | --- |
| **Response** | Acknowledged. Figure 2 was created using Datawrapper (https://www.datawrapper.de), an online data visualisation tool that uses Natural Earth as its base map layer. The Figure 2 legend has been updated to include: (a) a direct link to the Datawrapper platform and the Natural Earth base layer (https://www.naturalearthdata.com), and (b) a statement confirming that the Natural Earth base layer data are in the public domain and freely available for use without restriction (https://www.naturalearthdata.com/about/terms-of-use/). These details are now included in the figure legend for Figure 2 in the manuscript. |

**Comment 5: Data Availability Statement**

| **Editor's Comment** | *All PLOS journals now require all data underlying the findings to be freely available. Please explain if data cannot be made publicly available for ethical or legal reasons.* |
| --- | --- |
| **Response** | Acknowledged. A Data Availability Statement has been added to the manuscript. All data supporting the findings of this review are available within the manuscript and its supplementary materials. The extracted prevalence data and study characteristics are provided in the supplementary tables (S1 Table and S2 Table). No individual-level or patient-identifiable data were used in this review; all data are derived from published studies and are therefore publicly available: **Lines 1302-1349.** |

**Comment 6: Financial Disclosure Statement**

| **Editor's Comment** | *Please amend your detailed Financial Disclosure statement with: (1) author initials alongside each funding source, (2) funder role statement, and (3) any salary information.* |
| --- | --- |
| **Response** | Acknowledged. The Funding statement has been revised to include: (1) author initials indicating which authors received support from which funding source; (2) a statement clarifying the funder's role (the funder had no role in study design, data collection and analysis, decision to publish, or preparation of the manuscript); and (3) a statement that no salary costs were covered by the study grant for any author beyond standard institutional arrangements. The revised statement is in the Funding section (**end of manuscript lines 1510–1515**). |

**Comment 7: Systematic Review Data Requirements**

| **Editor's Comment** | *As required by our policy on Data Availability, please ensure your manuscript or supplementary information includes: (1) A numbered table of all studies identified in the literature search with exclusion reasons; (2) A table of all data extracted from primary sources including extractor names, dates, eligibility confirmation, and all extracted data; (3) Risk of bias assessments for each study; (4) Explanation of how missing data were handled.* |
| --- | --- |
| **Response** | (1) A numbered table of all studies identified in the literature search, including exclusion reasons at each stage, has been added as S2 Table in the supplementary material.  (2) A comprehensive data extraction table — including extractor names (NH and UUN), extraction dates, eligibility confirmation, and all extracted data fields (country, year, study design, setting, geographic scope, sample size, number of cases, and prevalence with 95% CI) — has been provided as S1 Table in the supplementary material.  (3) Risk of bias assessments for each included study, conducted using the Newcastle-Ottawa Scale, are provided in S3 Table in the supplementary material. A brief summary of quality assessment findings has also been added to the Study Eligibility and Quality Assessment subsection of Methods.  (4) An explanation of how missing data were handled has been added to the Data Extraction and Statistical Analyses subsection of Methods. In brief, no imputation of missing data was performed. Where studies did not report 95% confidence intervals, these were calculated from reported numerators and denominators using the Wilson score method prior to pooling. |

**REVIEWER #2**

**Methods Comments**

***Comment 2.1: Geographic Scope and Search Strategy***

| **Reviewer's Comment** | *The search strategy does not clearly specify how the geographical scope was operationalized. The absence of an explicit definition of included countries and how this regional scope was implemented limits reproducibility. The search appears to have been conducted up to 2023, and clarification is needed as to whether it was updated prior to submission.* |
| --- | --- |
| **Response** | We thank the reviewer for this methodological point. We have addressed it in two ways:  First, we have explicitly defined the geographic scope using the United Nations Statistics Division M49 standard geoscheme for Eastern Africa, listing all 20 countries included within this classification. This definition is now stated clearly in the Database Searches subsection of Methods and explains precisely how the regional restriction was applied at both the database search stage and the screening stage.  Second, we confirm that the literature search was updated in March 2026 prior to submission, covering the period January 2024 to March 2026 across all seven original databases. This is stated in the Database Searches subsection and the Results section.  Database Searches subsection of Methods **pages 5–6** : **Lines 292–325.** |

***Comment 2.2: Statistical Methods Transparency***

| **Reviewer's Comment** | *The type of random-effects model and heterogeneity statistics are reported in Results but should be more clearly described in Methods. Clarification is needed regarding the data transformation applied, the specification of the random-effects model, and the approach used to assess heterogeneity.* |
| --- | --- |
| **Response** | We thank the reviewer for identifying this gap. The Data Extraction and Statistical Analyses subsection of Methods has been substantially expanded to provide full methodological transparency. The revised section now explicitly describes: (1) the Freeman-Tukey Double Arcsine Transformation (FTDAT) applied prior to pooling, with justification for its use when proportions approach 0 or 1 or when sample sizes vary substantially; (2) the random-effects model specification using the DerSimonian-Laird method-of-moments estimator for between-study variance (τ²), implemented via STATA's metaprop command; (3) the approach to quantifying between-study heterogeneity using I² and Cochran's Q statistic with conventional interpretation thresholds (25%, 50%, 75% for low, moderate, and high heterogeneity); and (4) the use of funnel plots to assess potential publication bias, with explanation of why formal statistical testing (Egger's test) was not applied given the small number of studies.  **Manuscript location: Data Extraction and Statistical Analyses: Points (1)–(3)–line 427–462, and point (4): Results section, funnel plot interpretation paragraph –line 772–776.** |

***Comment 2.3: Grey Literature Search Methods***

| **Reviewer's Comment** | *The methods do not specify how grey literature was identified (e.g., search terms used, screening approach, number of results reviewed, or limits applied).* |
| --- | --- |
| **Response** | We thank the reviewer for this observation. A dedicated Grey Literature Search subsection has been added to Methods, detailing: (1) the sources searched; (2) the search terms used, which were identical to those applied in the structured database search; (3) the approach for Google Scholar, where the first 200 results were screened following established practice in systematic reviews; (4) the screening approach, in which the same two independent reviewers conducted abstract-level screening of all grey literature records; and (5) confirmation that the grey literature search was also updated in March 2026 to cover the period January 2024 to March 2026.  Manuscript location: Grey Literature Search subsection of Methods runs from **line 327 to line 376.** |

**Results Comments**

***Comment 2.4: PRISMA Flow Diagram Clarity***

| **Reviewer's Comment** | *The PRISMA flow diagram follows the expected structure; however, a clearer and more visually standardized version consistent with PRISMA 2020 presentation formats would improve readability.* |
| --- | --- |
| **Response** | Acknowledged. Both PRISMA flow diagrams (Figure 1 for prevalence studies and Figure 8 for DALY/economic burden studies, as per the separate flowcharts now provided in response to Reviewer 3 Comment 3.1) have been redrawn to comply fully with the PRISMA 2020 format, including standardized box shapes, consistent fonts, clear counts at each stage, and the correct node structure distinguishing records identified through database searching, additional records identified through other sources, records after duplicates removed, records screened, full-text articles assessed for eligibility, and studies included. The updated figures are provided as separate .tif files but also appear in the manuscript on pages: **Figure 1** (PRISMA Flow Diagram: Prevalence Studies): **page 14**, **Figure 8** (PRISMA Flow Diagram: Economic Burden Studies) **page 35**. |

***Comment 2.5: Geographic Contribution Clarity***

| **Reviewer's Comment** | *The relationship between countries mentioned in the text, those displayed in Figure 2, and those reported in Table 1 is not always clear. Reporting the corresponding references immediately after each country name would help.* |
| --- | --- |
| **Response** | We thank the reviewer for this suggestion. We have revised the relevant text in the Results section so that each country named in the narrative is followed immediately by the relevant study reference(s) in parentheses. For example, references for Ethiopia, Kenya, Uganda, and Rwanda studies are now cited directly after the country name at each occurrence in the results narrative. Table 1 and Figure 2 have also been made consistent with each other and with the text: Figure 2 now shows only the four countries in which studies were conducted (Ethiopia, Kenya, Uganda, Rwanda), with Burundi and Tanzania removed. Page 16 of the manuscript. |

***Comment 2.6: Descriptive Guidance for Pooled Estimate***

| **Reviewer's Comment** | *Although individual prevalence estimates are reported in tables, additional descriptive guidance in the Results would help contextualize the pooled estimate and facilitate interpretation of the forest plot.* |
| --- | --- |
| **Response** | We agree and have expanded the results narrative to provide richer contextualisation of the pooled estimate and its interpretation. The revised Results section now: (1) explicitly describes the range of individual study prevalence estimates and explains why they vary so substantially; (2) explains the meaning of the transformed pooled estimate (1.19, 95% CI: 1.14–1.25) and provides guidance on its back-transformation; (3) identifies the key features of the forest plot, including the minimal overlap of confidence intervals between Ethiopian and non-Ethiopian studies, the correspondence between CI width and study precision, and the positioning of the pooled diamond; and (4) contextualises the pooled estimate against the Ethiopia-specific (4.52%) and non-Ethiopian (0.20%) subgroup estimates, making clear that the overall pooled figure should not be interpreted as a single representative regional prevalence.  **Manuscript location: Results section, paragraphs describing the forest plot (Figure 4) and pooled prevalence interpretation: pages 20–21, lines 667-721.** |

***Comment 2.7: Forest Plot Labeling***

| **Reviewer's Comment** | *The forest plot would benefit from clearer labeling, including the addition of references or unique study identifiers alongside study labels.* |
| --- | --- |
| **Response** | We thank the reviewer for this suggestion. The forest plots (Figures 4, 6, and 8) have been updated to include reference numbers alongside each study label, consistent with the reference list in the manuscript. For example, study labels now appear as 'Alemu et al., 2011 [30]', 'Geshere Oli et al., 2012 [12]', 'Deribe et al., 2019 [20]', and so on, allowing readers to locate the full citation directly from the forest plot without cross-referencing the study characteristics tables. The updated figures are provided as separate .tif files.  **Manuscript location: Figures 4 (page 21), 6 (page 26), and 8 (page 32).** |

***Comment 2.8: Funnel Plot Description***

| **Reviewer's Comment** | *The funnel plot is presented but only limited descriptive information is provided. A brief statement describing the main visual pattern would guide interpretation.* |
| --- | --- |
| **Response** | We thank the reviewer for this observation. Detailed narrative descriptions of both funnel plots (Figures 5 and 7) have been added to the Results. For Figure 5 (full 14-study meta-analysis), the description now explains the asymmetric distribution of studies around the pooled estimate, the clustering of large precise studies near the apex, the rightward displacement of smaller Ethiopian studies reporting higher prevalence, and the leftward positioning of Uganda, Rwanda, and Kenyan nationwide surveys — and clarifies that this pattern reflects genuine epidemiological heterogeneity rather than publication bias. A parallel description is provided for Figure 7 (Ethiopia-only subgroup funnel plot).  Manuscript location: Results section, paragraphs following Figures 5(page 22) and 7(page 28). |

***Comment 2.9: Figure 5 Clarity***

| **Reviewer's Comment** | *Figure 5 is difficult to interpret. The legend and symbols are insufficiently explained, and it is not clear whether the figure summarizes included studies on DALYs, excluded economic studies, or provides a broader bibliometric overview.* |
| --- | --- |
| **Response** | We thank the reviewer for identifying this lack of clarity. What was previously Figure 5 was in fact the PRISMA flow diagram for the DALY and economic burden search arm of the review. Following the separation of PRISMA flow diagrams into two distinct figures in response to Comment 3.1 (Reviewer 3), this diagram is now presented as a clearly labelled and standalone figure (Figure 8 in the revised manuscript: 'PRISMA 2020 Flow Diagram: DALYs and Economic Burden Studies'). The figure legend has been rewritten to explicitly state that this diagram depicts the screening and selection process for the DALY and economic burden objective, and all symbols and decision nodes are now clearly labelled in PRISMA 2020 format(**Page 35**). |

**Conclusions Comments**

***Comment 2.10: Incomplete Geographical Coverage***

| **Reviewer's Comment** | *It would be helpful to clearly state that prevalence data are available from only a subset of countries within the region, with many countries not represented. Explicitly acknowledging this incomplete geographical coverage would help readers better understand the scope and limitations of the pooled estimate.* |
| --- | --- |
| **Response** | We agree and have made explicit statements to this effect in multiple sections. In the Results, we now state directly that prevalence studies were identified in only 4 of the 20 UN M49 Eastern Africa countries (Ethiopia, Kenya, Uganda, and Rwanda), representing 15% of the defined region, and that 16 countries in the classification had no eligible prevalence data. This is noted in the paragraph following the PRISMA diagram. In the Limitations subsection, incomplete geographic coverage is listed as a primary limitation. In the Conclusion, the absence of data from 16 of 20 East African countries is cited as a critical gap requiring urgent attention.  Manuscript location: Results section: Lines 516–522, page 16, Limitations subsection Lines 1448–1469, page 40 and Conclusion Lines 1472 onwards, page 40. |

***Comment 2.11: Epidemiological Variation Across Countries***

| **Reviewer's Comment** | *Substantial differences in prevalence across included countries likely reflect meaningful epidemiological variation driven by environmental exposure, population characteristics, and local contexts. Highlighting these aspects would strengthen interpretation and public health relevance.* |
| --- | --- |
| **Response** | We agree and have expanded the discussion of between-country epidemiological variation in the Discussion section. The revised Discussion now explicitly addresses how differences in volcanic soil composition and mineral content, rainfall patterns, barefoot exposure rates, footwear usage, and occupational characteristics across the four included countries plausibly explain the observed prevalence differences. We also note that Rwanda's lower prevalence is consistent with its nationwide prevention initiatives including mandatory footwear policies, and that Uganda's restricted burden aligns with its geographically limited areas of volcanic soil suitability. Additionally, the Discussion now addresses the findings in the context of the environmental suitability predictions from Deribe et al. (2020) as requested in Comment 3.10.  Manuscript location:  **Country-level epidemiological variation (Uganda, Rwanda, footwear policies):**   - **Lines 1330–1345, page 38** — paragraphs on Uganda's geographically restricted burden (highland volcanic areas) and Rwanda's lower prevalence in the context of nationwide prevention initiatives including mandatory footwear policies   **Broader prevalence heterogeneity and epidemiological variation across countries:**   - **Lines 1349–1369, page 39** — paragraph discussing the 20-fold difference between Ethiopian and non-Ethiopian pooled estimates and the drivers of between-country variation   **Environmental suitability predictions from Deribe et al. (2020):**   - **Lines 1394–1408, page 40** — paragraph contextualising findings against the environmental suitability modelling, citing Deribe et al. (2020) and the 114.5 million people in suitable areas across East Africa |

**Editorial and Presentation Comments**

***Comment 2.12: Figure Improvements***

| **Reviewer's Comment** | *Visual standardization of PRISMA flow diagram (Figure 1), addition of legend to Figure 2, inclusion of references in Figure 3, and clearer legend for Figure 5 would improve readability.* |
| --- | --- |
| **Response** | All figure improvements have been made: (1) both PRISMA flow diagrams now conform to PRISMA 2020 format (see Comment 2.4); (2) Figure 2 (map of podoconiosis prevalence studies) now includes a legend indicating the number of studies per country and the country names, and Burundi and Tanzania have been removed (see Comments 3.7 and 3.12); (3) forest plot figures now include reference numbers alongside study labels (see Comment 2.7); and (4) the previously unclear Figure 5 has been clarified as the DALY/economic burden PRISMA flow diagram (see Comment 2.9). All updated figures are submitted as separate .tif files. |

***Comment 2.13: Table 1 Formatting***

| **Reviewer's Comment** | *Minor formatting improvements could be made to Table 1, such as standardizing the labeling of the 'Author' column.* |
| --- | --- |
| **Response** | Acknowledged. The 'Author' column in Table 1 has been standardised to use consistent formatting throughout (bold author names with year in parentheses, using the same format as citations in the text). Any anomalous date or timestamp entries in the table (also noted in Comment 3.14) have been removed. Edits are available from **page 18.** |

***Comment 2.14: Full Search Strategies***

| **Reviewer's Comment** | *Providing the full search strategies used for each database, ideally as supplementary material, would be helpful and align with PRISMA 2020 recommendations.* |
| --- | --- |
| **Response** | Acknowledged. The full search strategies for all electronic databases (PubMed/MEDLINE, EMBASE, SCOPUS, Web of Science, EconLit, WHO AFROLIB, and Google Scholar) — for both the prevalence arm and the DALY/economic burden arm of the review — have been provided in S4 Table as supplementary material. This is now referenced in the Database Searches subsection of Methods. |

**REVIEWER #3**

**Methods Comments**

***Comment 3.1: Separate PRISMA Flowcharts***

| **Reviewer's Comment** | *Since there are two objectives, the authors should provide two PRISMA flowcharts — one for prevalence and another for DALYs.* |
| --- | --- |
| **Response** | We thank the reviewer for this structural point and we agree entirely. Two separate PRISMA 2020 flow diagrams are now provided: Figure 1(**page 14-16**) depicts the screening and selection process for the prevalence objective (yielding 14 included studies), and Figure 8(**page 35**) depicts the screening and selection process for the DALYs and economic burden objective (yielding 1 included study). Both diagrams conform to the PRISMA 2020 format and are submitted as separate .tif files. The narrative in the Results section has been updated to refer to each diagram correctly. |

***Comment 3.2: Ethiopia-Specific Analysis***

| **Reviewer's Comment** | *Ethiopia had the highest number of studies and consistently showed prevalence over 2.79%. Combining with other East African countries lowered the pooled estimate to 0.04%. Calculate pooled estimates for Ethiopia separately and present results both with and without Ethiopia for the East African region.* |
| --- | --- |
| **Response** | We thank the reviewer for this suggestion, which we have fully implemented. The revised Results section now presents three meta-analytic estimates: (1) the full 14-study pooled estimate across all four countries (overall pooled prevalence: 1.19, 95% CI: 1.14–1.25 on the transformed scale); (2) the Ethiopia-specific subgroup pooled estimate (k=10 studies; 4.52%, 95% CI: 3.92–5.16%); and (3) the non-Ethiopian subgroup pooled estimate (k=4 studies from Kenya, Uganda, and Rwanda; 0.20%, 95% CI: 0.10–0.33%). Each subgroup is presented with its corresponding forest plot (Figures 6 and 8 respectively) and funnel plot (Figure 7 for Ethiopia). The more than 20-fold difference between the Ethiopian and non-Ethiopian estimates is discussed explicitly as the most important finding of the heterogeneity analyses.  Regarding the mention of a 0.04% estimate in the reviewer's comment: as clarified in the response to Comment 3.4 below, this figure arose from a transcription error in the original submission. The correct back-transformed pooled prevalence is addressed in that response.  Manuscript location: Results section  **Full 14-study pooled estimate (1.19, 95% CI: 1.14–1.25):**   - **Lines 714–721, page 21** (forest plot and pooled estimate interpretation paragraph, Figure 4)   **Ethiopian subgroup analysis (4.52%, 95% CI: 3.92–5.16%):**   - **Lines 883–925, pages 26–27** — narrative description of the Ethiopian subgroup analysis - **Line 926, page 27** — Figure 6 (Forest Plot: Ethiopia, k=10 studies) - **Line 961, page 28** — Figure 7 (Funnel Plot: Ethiopia, k=10 studies)   **Non-Ethiopian subgroup analysis (0.20%, 95% CI: 0.10–0.33%):**   - **Lines 1181–1199, page 32** — introductory paragraph for the non-Ethiopian subgroup - **Line 1196, page 32** — Figure 8 (Forest Plot: East Africa Excluding Ethiopia, k=4 studies) - **Lines 1201–1228, page 33** — narrative interpretation of the non-Ethiopian pooled estimate   **The more than 20-fold difference explicitly stated:**   - **Lines 1240–1244, page 34** — synthesising paragraph explicitly stating the 20-fold difference as the key finding of the heterogeneity analyses. |

***Comment 3.3: Data Extraction Variables***

| **Reviewer's Comment** | *The authors should describe the parameters or variables extracted into the Microsoft Excel sheet in the main paper itself (the supplementary information is not mentioned).* |
| --- | --- |
| **Response** | Acknowledged. The Data Extraction and Statistical Analyses subsection of Methods has been revised to explicitly list the variables extracted from each included study directly in the main text: study author and year, country, study design, sample size, number of confirmed cases, and prevalence with 95% CI (**lines 428–434, page 12**). Additionally, a reference to the supplementary data extraction table (S1 Table) has been added here. The supplementary material is now explicitly mentioned and described in the Methods.  Manuscript location: Data Extraction and Statistical Analyses subsection of Methods. |

**Results Comments**

***Comment 3.4: Figure 3 Text Discrepancy***

| **Reviewer's Comment** | *There is a discrepancy between the figure and text. Figure 3 shows the model estimate of 1.19 (95% CI 1.14–1.25), but the text mentions 0.04% (95% CI 0.02–0.05%). The authors need to recheck or explain this.* |
| --- | --- |
| **Response** | We thank the reviewer for identifying this discrepancy and sincerely apologise for the confusion. The value of 1.19 (95% CI: 1.14–1.25) shown in the forest plot is the pooled prevalence on the Freeman-Tukey double arcsine transformed scale, which is not directly interpretable as a percentage. When back-transformed to the original proportion scale, this corresponds to a pooled prevalence of approximately 4.3% across all 14 studies. The figure of '0.04%' cited in the original text was an error introduced during drafting and did not correspond to any analysis output. This has been corrected throughout the manuscript. The Results section now clearly explains the distinction between the transformed pooled estimate (1.19) and its back-transformed interpretation, and no figure of 0.04% appears in the revised manuscript.  Manuscript location: Results section  The relevant content appears in three locations across the manuscript:  **Abstract (transformed pooled estimate first stated):**   - **Lines 57–58, page 2** — "The overall pooled prevalence was 1.19 (95% CI: 1.14–1.25) on the transformed scale"   **Methods (back-transformation procedure described):**   - **Lines 446–447, page 12** — "Back-transformation of pooled estimates to the original proportion scale was performed following meta-analysis to aid interpretability"   **Results (key correction — distinction between transformed estimate and back-transformed interpretation):**   - **Lines 714–721, page 21** — the paragraph beginning "The pooled prevalence of 1.19 (95% CI: 1.14–1.25) on the transformed scale, back-transformed to a prevalence proportion for interpretation..." — this is the primary location where the distinction is explained and where the erroneous 0.04% figure has been removed. |

***Comment 3.5: Heterogeneity Analysis***

| **Reviewer's Comment** | *The authors fail to describe the heterogeneity driving the funnel plot. What factors are contributing to wide variation even though most studies are from Ethiopia? Please conduct additional subgroup analyses.* |
| --- | --- |
| **Response** | We thank the reviewer for this critical point. We have substantially expanded the heterogeneity analyses and their interpretation in the revised manuscript. In addition to the Ethiopia-specific and non-Ethiopian subgroup analyses (see Comment 3.2), we have conducted a stratified analysis by sample size (studies with N<1,000 vs. N≥1,000), which reveals that the inverse relationship between sample size and observed prevalence is a consistent structural feature of this literature. Smaller studies systematically targeted confirmed endemic foci (yielding higher prevalence estimates with wider confidence intervals), while larger studies captured broader geographic sampling frames including both endemic and non-endemic areas (yielding lower, more precise estimates). This pattern persists within Ethiopia (I²=97.8% even in the Ethiopia-only subgroup), indicating that country-level factors alone do not account for the observed heterogeneity. The residual within-Ethiopia heterogeneity is attributed to genuine subnational variation in disease intensity driven by differences in altitude, volcanic soil distribution, barefoot exposure rates, and access to preventive interventions across districts. All these analyses are now described in dedicated subsections with accompanying tables (Table 3) and the relevant forest and funnel plots.  Manuscript location: Results section (Heterogeneity and Subgroup Analyses, including Table 3 and Figures 5–8).  **Stratified analysis by sample size (N<1,000 vs. N≥1,000) — introductory paragraph:**   - **Lines 659–665, page 19** — paragraph beginning "Stratifying the 14 included studies by sample size threshold..." introducing the inverse relationship between sample size and prevalence   **Table 3** (Study characteristics stratified by sample size):   - **Line 1045, page 30**   **Group A: Small Studies (N<1,000):**   - **Line 1137, page 31**   **Group B: Large Studies (N≥1,000):**   - **Line 1154, page 31**   **I²=97.8% within Ethiopia and subnational heterogeneity:**   - **Lines 915–922, page 27** — paragraph reporting the persisting heterogeneity within the Ethiopia-only subgroup (I²=97.8%) and attributing residual variation to differences in altitude, volcanic soil distribution, barefoot exposure rates, and access to preventive interventions   **Synthesising paragraph linking sample size stratification to Ethiopian evidence:**   - **Lines 1036–1043, page 30** — paragraph beginning "a parallel pattern emerges when studies are stratified by sample size rather than setting..." |

***Comment 3.6: Country Percentage Discrepancy***

| **Reviewer's Comment** | *The paper states 65% of studies are from Ethiopia, but 10/14 gives 71% (per Figure 2).* |
| --- | --- |
| **Response** | The reviewer is correct. This was an error in the original manuscript. The text has been corrected throughout to state that Ethiopia contributed 10 of 14 included studies, representing approximately 71% of the total. The figure of 65% has been removed from all instances in the revised manuscript.  Manuscript location:  **Results section — paragraph following Figure 1 (PRISMA diagram):**   - **Lines 516–522, page 16** — "Ethiopia had the highest number of studies (n=10 studies), representing about 71% of the total number of studies included in this review"   **Limitations subsection:**   - **Lines 1457–1458, page 40** — "The evidence base is heavily skewed toward Ethiopia, which contributed 71% of included studies" |

***Comment 3.7: Country List Inconsistency***

| **Reviewer's Comment** | *The text mentions Burundi and Tanzania, but the figure does not show them.* |
| --- | --- |
| **Response** | The reviewer is correct. The mention of 'Burundi and Tanzania' in the Results section was an error retained from an earlier draft and did not correspond to any included studies. No studies from Burundi or Tanzania met the inclusion criteria. The text has been corrected to remove these country names from the list of countries represented in the included studies. Correspondingly, Burundi and Tanzania have been removed from Figure 2 (the map of podoconiosis prevalence studies) as noted in Comment 3.12. The figure and text are now consistent.  Manuscript location:  **Results section — paragraph following Figure 1:**   - **Lines 516–522, page 16** — this is where the tracked deletion of "Burundi and Tanzania" appears, in the sentence listing the countries represented in the included studies. The strikethrough of "Burundi and Tanzania" is visible in the track-changes version at line 522.   **Figure 3** (Map of Podoconiosis Prevalence Studies in East Africa):   - **Page 16** — the map immediately follows this paragraph and has had Burundi and Tanzania removed accordingly. |

***Comment 3.8: Single DALY Study – Meta-analysis Question***

| **Reviewer's Comment** | *For DALYs, only one study is included. Does this qualify as a meta-analysis? Suggestion to split into two papers or use meta-analysis only for prevalence component.* |
| --- | --- |
| **Response** | This is a valid point; we agree that a meta-analysis cannot be conducted on a single study, and we have revised the manuscript accordingly. The title and framing of the paper have been updated to clarify that the meta-analytic component applies exclusively to the prevalence objective, while the DALYs and economic burden objective is framed as a systematic review of available evidence. The revised title will now read: 'The Health and Economic Burden of Podoconiosis in East Africa: A Systematic Review and Meta-Analysis of Prevalence.' The DALYs section now clearly presents the findings from Deribe et al. (2020) as the single available source of DALY and economic burden data for the region and explicitly acknowledges that no pooling was possible. We have opted not to split the paper into two publications, as the prevalence and burden objectives are intrinsically linked and the combined framing strengthens the case for more comprehensive burden research.  Manuscript location: Title, Abstract (Methods sentence), and DALYs/Economic Burden Results subsection. |

**Discussion Comments**

***Comment 3.9: Justify 'Neglected' Claim with Data***

| **Reviewer's Comment** | *The authors need to explain why podoconiosis is neglected among NTDs. Provide data on DALYs for other NTDs and podoconiosis, as well as funding amounts allocated. Support these points with data in both introduction and discussion.* |
| --- | --- |
| **Response** | We thank the reviewer for pushing us to substantiate this claim more rigorously. The Introduction and Discussion have been revised to include comparative data supporting the characterisation of podoconiosis as neglected even within the NTD category. Specifically: (1) In the Introduction, we now cite the 2015 GBD estimates for comparator NTDs — soil-transmitted helminthiases (~4,443 thousand DALYs), schistosomiasis (~3,514 thousand), dengue fever (~2,610 thousand), and lymphatic filariasis (~2,071 thousand) — against which podoconiosis cannot even be directly compared given its continued absence from the GBD framework, a disparity that points strongly to systematic underestimation rather than genuinely lower impact. (2) In the Discussion, we reinforce this by noting that the 2023 WHO global NTD report tracked DALY reductions for 16 NTDs but had no estimates for podoconiosis, and that only Ethiopia has any modelled DALY estimates (172,073 DALYs/year; Deribe et al., 2020). Data on podoconiosis-specific research funding are not systematically available in the published literature; we have stated this limitation rather than speculating.  Manuscript location:  **(1) Introduction — GBD comparator NTD DALY estimates:**   - **Lines 215–225, page 7** — the paragraph beginning "Podoconiosis is a disease with substantial and quantifiable burden..." which cites the 2015 GBD estimates for soil-transmitted helminthiases (~4,443 thousand DALYs), schistosomiasis (~3,514 thousand), dengue fever (~2,610 thousand), and lymphatic filariasis (~2,071 thousand), and makes the case that podoconiosis's absence from the GBD points to systematic underestimation (lines 219–225 specifically)   **(2) Introduction — 2023 WHO NTD report and 16 NTDs:**   - **Lines 227–232, page 7** — the paragraph noting that the 2023 global NTD report tracked DALY reductions for 16 NTDs but lacked estimates for podoconiosis   **(2) Discussion — evidence gaps and underinvestment:**   - **Lines 1372–1383, page 39** — the paragraph beginning "The burden analyses reveal an equally stark evidence gap..." which reinforces the point about underinvestment, references the single Ethiopian DALY estimate of 172,073 DALYs/year (Deribe et al., 2020), and cites the disease's exclusion from the GBD as a driver of continued neglect |

***Comment 3.10: Address Predictive Model from Introduction***

| **Reviewer's Comment** | *The authors don't address their findings in relation to the predictive model described in the introduction, which would be beneficial.* |
| --- | --- |
| **Response** | We agree and have added a dedicated paragraph in the Discussion that explicitly contextualises our prevalence findings against the environmental suitability model of Deribe et al. (2020). This paragraph notes that the suitability model predicted podoconiosis-conducive conditions in 11 East African countries, with over 90 million people at risk in the region, yet our review identified prevalence data from only 4 of those countries. We discuss this discrepancy as evidence that current prevalence estimates substantially underrepresent the true burden, and that environmental suitability mapping should actively guide future surveillance priorities rather than serve merely as background context.  Manuscript location: Discussion section: **Lines 1394–1408, page 40** |

***Comment 3.11: Shorten Discussion***

| **Reviewer's Comment** | *The discussion section can be shortened.* |
| --- | --- |
| **Response** | Acknowledged. The Discussion has been reviewed for concision. Repetitive passages that restated findings already covered in Results have been removed or consolidated. The overall Discussion has been streamlined while ensuring that all substantive responses to reviewer comments are retained. Key additions requested by reviewers (country-level epidemiological variation, comparison with predictive models, justification of the 'neglected' characterisation) are incorporated as efficiently as possible without redundancy. |

**Editorial Comments**

***Comment 3.12: Figure 2 – Show Study Numbers***

| **Reviewer's Comment** | *Figure 2, the number of studies can be shown on the map. Remove Burundi and Tanzania from the map.* |
| --- | --- |
| **Response** | Both changes have been made. Figure 2 (the map of podoconiosis prevalence studies in East Africa) has been updated to: (a) display the number of included studies for each of the four represented countries (Ethiopia: n=10; Kenya: n=2; Uganda: n=1; Rwanda: n=1), annotated directly on the map; and (b) remove Burundi and Tanzania, which were erroneously included in the original figure but had no included prevalence studies. The updated figure is submitted as a separate .tif file. |

***Comment 3.13: Figure 4 – Label Funnel Plot Points***

| **Reviewer's Comment** | *Figure 4: The dots in the funnel chart should be labelled with the studies.* |
| --- | --- |
| **Response** | Acknowledged. The funnel plots (Figures 5 and 7) have been updated to label each data point with the first author's surname and year of publication (e.g., 'Deribe 2019', 'Geshere Oli 2012'). This allows readers to identify individual studies within the funnel plots without cross-referencing the forest plots. The updated figures are also submitted as separate .tif files. |

***Comment 3.14: Table 1 – Remove Dates/Timestamps***

| **Reviewer's Comment** | *Table 1 contains dates and timestamps in two rows that need to be removed.* |
| --- | --- |
| **Response** | Acknowledged. The erroneous dates and timestamps appearing in two rows of Table 1 have been identified and removed. The affected cells have been corrected to display only the appropriate study characteristic data. The revised Table 1 has been checked in full for formatting consistency. |

***Comment 3.15: Rewrite Introduction DALYs Paragraphs***

| **Reviewer's Comment** | *The paragraphs in the introduction mentioning 57 million DALYs, 22 million DALYs, and global burden of disease are very confusing. Rewrite for clear narrative flow and logic.* |
| --- | --- |
| **Response** | We thank the reviewer for this feedback. The relevant paragraphs in the Introduction have been rewritten to present the burden of NTDs more clearly and with a more logical narrative arc. The revised text now: (1) first establishes that NTDs disproportionately affect sub-Saharan Africa and cause substantial morbidity rather than mortality; (2) then introduces the global DALY burden of NTDs with the 57 million figure in context (sub-Saharan Africa, annually); (3) then places this in the global perspective with the 22 million figure (globally, 2012), clarifying that NTDs account for approximately 40% of malaria's DALYs; and (4) then makes the specific point that podoconiosis is absent from the GBD framework, making it impossible to include it in any of these calculations, which is the central justification for this review. The revised paragraphs are now presented in a clear, logical sequence.  Manuscript location:  **Introduction: (1) & (2) First paragraph — NTDs, morbidity vs. mortality, 57 million DALYs (sub-Saharan Africa) and 22 million DALYs (globally, 2012):**   - **Lines 172–188, page 6** — the paragraph beginning "On the other hand, several NTDs, such as podoconiosis, do not result in fatalities...", which establishes that NTDs cause substantial morbidity rather than mortality, introduces the 57 million DALYs figure for sub-Saharan Africa annually (line 176), and then places this in global perspective with the 22 million figure (line 182–183), clarifying that NTDs account for approximately 40% of malaria's DALYs   **(3) & (4) Second paragraph — podoconiosis absent from GBD, comparator NTD DALY figures:**   - **Lines 203–225, page 7** — the paragraph beginning "Podoconiosis is a disease with substantial and quantifiable burden...", which makes the specific point that podoconiosis remains absent from the GBD framework (lines 203–210), provides the comparator NTD DALY estimates (lines 219–222), and concludes that this disparity points to systematic underestimation of the podoconiosis burden (lines 222–225) |

We hope that the revised manuscript and this point-by-point response adequately address all concerns raised by the Editor and Reviewers. We are grateful for the constructive and detailed feedback, which has substantially strengthened the manuscript.

Sincerely,

**Nadia HITIMANA and co-authors**
