# Supplementary material for: The health and economic burden of podoconiosis in East Africa: A systematic review and meta-analysis of health outcomes with narrative synthesis of economic evidence
Source: PLoS Negl Trop Dis. 2026 Jun 17;20(6):e0014427. doi: 10.1371/journal.pntd.0014427 (PMC13340807; doi:10.1371/journal.pntd.0014427)
Supplement: Peer review history

# Peer review: Decision Letter 0

**DOI:** 10.1371/journal.pntd.0014427.r001

**Authors:** Maria Victoria Periago

11 Feb 2026

PNTD-D-25-01336

The Health and Economic Burden of Podoconiosis in East Africa: A Systematic Review and Meta-Analysis

PLOS Neglected Tropical Diseases

Dear Dr. Hitimana,

Thank you for submitting your manuscript to PLOS Neglected Tropical Diseases. After careful consideration, we feel that it has merit but does not fully meet PLOS Neglected Tropical Diseases's publication criteria as it currently stands. Therefore, we invite you to submit a revised version of the manuscript that addresses the points raised during the review process.

Please submit your revised manuscript within by Apr 12 2026 11:59PM. If you will need more time than this to complete your revisions, please reply to this message or contact the journal office at plosntds@plos.org. When you're ready to submit your revision, log on to https://www.editorialmanager.com/pntd/ and select the 'Submissions Needing Revision' folder to locate your manuscript file.

Please include the following items when submitting your revised manuscript:

* A letter that responds to each point raised by the editor and reviewer(s). You should upload this letter as a separate file labeled 'Response to Reviewers'. This file does not need to include responses to any formatting updates and technical items listed in the 'Journal Requirements' section below.

* A marked-up copy of your manuscript that highlights changes made to the original version. You should upload this as a separate file labeled 'Revised Manuscript with Track Changes'.

* An unmarked version of your revised paper without tracked changes. You should upload this as a separate file labeled 'Manuscript'.

If you would like to make changes to your financial disclosure, competing interests statement, or data availability statement, please make these updates within the submission form at the time of resubmission. Guidelines for resubmitting your figure files are available below the reviewer comments at the end of this letter.

We look forward to receiving your revised manuscript.

Kind regards,

María Victoria Periago

Section Editor

PLOS Neglected Tropical Diseases

María Periago

Section Editor

PLOS Neglected Tropical Diseases

Shaden Kamhawi

co-Editor-in-Chief

PLOS Neglected Tropical Diseases

orcid.org/0000-0003-4304-636XX

Paul Brindley

co-Editor-in-Chief

PLOS Neglected Tropical Diseases

orcid.org/0000-0003-1765-0002

**Additional Editor Comments:**

The authors have reported on the health and economic burden of podoconiosis in East Africa using a systematic review and meta-analysis of the prevalence and the objectives of the study are clearly stated. Nontheless, the reviewers have raised come important issues that need to be addressed.

**Journal Requirements:**

If the reviewer comments include a recommendation to cite specific previously published works, please review and evaluate these publications to determine whether they are relevant and should be cited. There is no requirement to cite these works unless the editor has indicated otherwise.

1) Please ensure that the CRediT author contributions listed for every co-author are completed accurately and in full.

At this stage, the following Authors/Authors require contributions: Nadia Hitimana, Vasso ANAGNOSTOPOULOU, Stephen BREMNER, Naillah UMUTONI UWIMANA, Natalia Hounsome, Lawrence RUGEMA, Leon MUTESA, and Maya SEMRAU. Please ensure that the full contributions of each author are acknowledged in the "Add/Edit/Remove Authors" section of our submission form.

The list of CRediT author contributions may be found here: https://journals.plos.org/plosntds/s/authorship#loc-author-contributions

2) Please upload all main figures as separate Figure files in .tif or .eps format. For more information about how to convert and format your figure files please see our guidelines:

https://journals.plos.org/plosntds/s/figures

3) We have noticed that you have uploaded Supporting Information files, but you have not included a list of legends. Please add a full list of legends for your Supporting Information files after the references list.

4) Some material included in your submission may be copyrighted. According to PLOSu2019s copyright policy, authors who use figures or other material (e.g., graphics, clipart, maps) from another author or copyright holder must demonstrate or obtain permission to publish this material under the Creative Commons Attribution 4.0 International (CC BY 4.0) License used by PLOS journals. Please closely review the details of PLOSu2019s copyright requirements here: PLOS Licenses and Copyright. If you need to request permissions from a copyright holder, you may use PLOS's Copyright Content Permission form.

Please respond directly to this email and provide any known details concerning your material's license terms and permissions required for reuse, even if you have not yet obtained copyright permissions or are unsure of your material's copyright compatibility. Once you have responded and addressed all other outstanding technical requirements, you may resubmit your manuscript within Editorial Manager.

Potential Copyright Issues:

i) Figure 2. Please (a) provide a direct link to the base layer of the map (i.e., the country or region border shape) and ensure this is also included in the figure legend; and (b) provide a link to the terms of use / license information for the base layer image or shapefile. We cannot publish proprietary or copyrighted maps (e.g. Google Maps, Mapquest) and the terms of use for your map base layer must be compatible with our CC BY 4.0 license.

Note: if you created the map in a software program like R or ArcGIS, please locate and indicate the source of the basemap shapefile onto which data has been plotted.

If your map was obtained from a copyrighted source please amend the figure so that the base map used is from an openly available source. Alternatively, please provide explicit written permission from the copyright holder granting you the right to publish the material under our CC BY 4.0 license.

If you are unsure whether you can use a map or not, please do reach out and we will be able to help you. The following websites are good examples of where you can source open access or public domain maps:

* U.S. Geological Survey (USGS) - All maps are in the public domain. (http://www.usgs.gov)

* PlaniGlobe - All maps are published under a Creative Commons license so please cite u201cPlaniGlobe, http://www.planiglobe.com, CC BY 2.0u201d in the image credit after the caption. (http://www.planiglobe.com/?lang=enl)

* Natural Earth - All maps are public domain. (http://www.naturalearthdata.com/about/terms-of-use/).

5) In the online submission form, you indicated that Data used in this review can be obtained upon request to the main author.. All PLOS journals now require all data underlying the findings described in their manuscript to be freely available to other researchers, either

1. In a public repository

2. Within the manuscript itself

3. Uploaded as supplementary information.

This policy applies to all data except where public deposition would breach compliance with the protocol approved by your research ethics board. If your data cannot be made publicly available for ethical or legal reasons (e.g., public availability would compromise patient privacy), please explain your reasons by return email and your exemption request will be escalated to the editor for approval. Your exemption request will be handled independently and will not hold up the peer review process, but will need to be resolved should your manuscript be accepted for publication. One of the Editorial team will then be in touch if there are any issues.

6) Please amend your detailed Financial Disclosure statement. This is published with the article. It must therefore be completed in full sentences and contain the exact wording you wish to be published.

1) State the initials, alongside each funding source, of each author to receive each grant. For example: "This work was supported by the National Institutes of Health (####### to AM; ###### to CJ) and the National Science Foundation (###### to AM)."

2) State what role the funders took in the study. If the funders had no role in your study, please state: "The funders had no role in study design, data collection and analysis, decision to publish, or preparation of the manuscript."

3) If any authors received a salary from any of your funders, please state which authors and which funders..

If you did not receive any funding for this study, please simply state: u201cThe authors received no specific funding for this work.u201d

7) As required by our policy on Data Availability, please ensure your manuscript or supplementary information includes the following:

- A numbered table of all studies identified in the literature search, including those that were excluded from the analyses.

- For every excluded study, the table should list the reason(s) for exclusion.

- If any of the included studies are unpublished, include a link (URL) to the primary source or detailed information about how the content can be accessed.

- A table of all data extracted from the primary research sources for the systematic review and/or meta-analysis. The table must include the following information for each study:

- Name of data extractors and date of data extraction

- Confirmation that the study was eligible to be included in the review.

- All data extracted from each study for the reported systematic review and/or meta-analysis that would be needed to replicate your analyses.

- If data or supporting information were obtained from another source (e.g. correspondence with the author of the original research article), please provide the source of data and dates on which the data/information were obtained by your research group.

- If applicable for your analysis, a table showing the completed risk of bias and quality/certainty assessments for each study or outcome.  Please ensure this is provided for each domain or parameter assessed. For example, if you used the Cochrane risk-of-bias tool for randomized trials, provide answers to each of the signalling questions for each study. If you used GRADE to assess certainty of evidence, provide judgements about each of the quality of evidence factor. This should be provided for each outcome.

- An explanation of how missing data were handled.

This information can be included in the main text, supplementary information, or relevant data repository. Please note that providing these underlying data is a requirement for publication in this journal, and if these data are not provided your manuscript might be rejected.

**Reviewers' Comments:**

Reviewer's Responses to Questions

**Key Review Criteria Required for Acceptance?**

As you describe the new analyses required for acceptance, please consider the following:

**Methods**

-Are the objectives of the study clearly articulated with a clear testable hypothesis stated?

-Is the study design appropriate to address the stated objectives?

-Is the population clearly described and appropriate for the hypothesis being tested?

-Is the sample size sufficient to ensure adequate power to address the hypothesis being tested?

-Were correct statistical analysis used to support conclusions?

-Are there concerns about ethical or regulatory requirements being met?

Reviewer #1: yes

Reviewer #2: The objectives of the study are clearly stated and directly address the research question of interest.

The study design (systematic review and meta-analysis of prevalence) is appropriate to address the stated objectives. The population of interest is generally well defined; however, the search strategy does not clearly specify how the geographical scope was operationalized (e.g. according to World Bank, WHO, UN, or other classifications). Although the manuscript later reports that studies were conducted across a defined number of countries, the absence of an explicit definition of the included countries and how this regional scope was implemented within the search strategy limits the reproducibility of the review.

Sample size considerations are inherently constrained by the available literature. Although the number of included studies is limited, this reflects the rarity of the condition rather than a limitation of the study design itself. Nonetheless, the small number of available studies further emphasizes the importance of an up-to-date literature search to ensure completeness. In this context, the search appears to have been conducted up to 2023, and clarification is needed as to whether the search was updated prior to submission. When applying the authors’ reported search strategy, additional potentially relevant studies appear to have been published more recently, suggesting that the review may not fully capture the most current evidence.

The statistical approach used to pool prevalence estimates is appropriate, including the use of a random-effects model and a variance-stabilizing transformation. While the type of random-effects model and heterogeneity statistics are reported in the Results, these methodological choices should be more clearly described in the Methods section. In particular, clarification is needed regarding the data transformation applied, the specification of the random-effects model, and the approach used to assess heterogeneity, in order to ensure transparency and reproducibility.

In addition, while the authors report searching Google Scholar, the methods do not specify how grey literature was identified (e.g. search terms used, screening approach, number of results reviewed, or limits applied), which further limits the transparency and reproducibility of the search strategy.

No concerns were identified regarding ethical or regulatory requirements, as the study is based exclusively on previously published data.

Reviewer #3: This is a systematic review and meta-analysis of the prevalence of Podoconiosis in East Africa. The methods used are suitable for the meta-analysis. Since there are two objectives, the authors should provide two PRISMA flowcharts—one for prevalence and another for DALYs. Ethiopia had the highest number of studies and consistently showed a prevalence of over 2.79%. Combining this with data from other East African countries has lowered the pooled estimate to 0.04%. It would be better if they calculated pooled estimates for Ethiopia separately and presented results both with and without Ethiopia for the East African region. The authors should describe the parameters or variables extracted into the Microsoft Excel sheet in the main paper itself (the supplementary information is not mentioned).

**********

**Results**

-Does the analysis presented match the analysis plan?

-Are the results clearly and completely presented?

-Are the figures (Tables, Images) of sufficient quality for clarity?

Reviewer #1: partially

Reviewer #2: The PRISMA flow diagram follows the expected structure; however, a clearer and more visually standardized version consistent with PRISMA 2020 presentation formats would improve readability and facilitate understanding of the study selection process.

The description of the geographical contribution of studies would benefit from greater clarity. The relationship between countries mentioned in the text, those displayed in Figure 2, and those reported in Table 1 is not always clear. Reporting the corresponding references immediately after each country name in the text would help readers more easily identify which studies originate from each setting.

The analyses presented are broadly consistent with the stated analysis plan, and the pooled prevalence estimate is reported together with confidence intervals and heterogeneity statistics (I² and Q-test). Although individual prevalence estimates are reported in tables, additional descriptive guidance in the Results (e.g. summarizing the range or variability of estimates) would help contextualize the pooled estimate and facilitate interpretation of the forest plot.

Regarding figures, the forest plot would benefit from clearer labeling, including the addition of references or unique study identifiers alongside study labels to improve readability. The funnel plot is presented in the Results; however, only limited descriptive information is provided in the text. A brief statement describing the main visual pattern observed would be sufficient to guide interpretation.

Finally, Figure 5 is difficult to interpret. The legend and symbols are insufficiently explained, and it is not clear whether the figure is intended to summarize included studies on DALYs, excluded economic evaluation studies, or to provide a broader bibliometric overview of the literature. In addition, the figure appears to include studies that fall outside the stated eligibility criteria (e.g. based on publication period), without this being explicitly clarified in the text or title. Clarifying the scope, purpose, and inclusion criteria underlying this figure, and improving its labeling and legend, would be necessary to avoid confusion and to justify its inclusion in the Results.

Overall, while the results appear technically sound, clearer textual description and improved integration between the narrative and the figures would strengthen the presentation and interpretation of the findings.

Reviewer #3: The results presented align with the analysis plan. However, there is a discrepancy between the figure and the text. Figure 3 shows the model estimate of 1.19 (95% CI 1.14-1.25), but the text mentions 0.04% (95% CI 0.02-0.05%). The authors need to recheck the results or provide an explanation for this. They also fail to describe the heterogeneity driving the funnel plot. What factors are contributing to this wide variation, even though most studies are from Ethiopia? Please conduct additional subgroup analyses to explain this phenomenon. The paper states 65% of studies are from Ethiopia, but 10/14 gives 71% (per figure 2). The text mentions Burundi and Tanzania, but the figure does not show them.

For DALYs, only one study is included. Does this qualify as a meta-analysis? I suggest that the authors split the paper into two parts and use meta-analysis only for the prevalence component, starting with the title. They could also consider writing one longer paper on prevalence and a separate shorter paper on DALYs.

**********

**Conclusions**

-Are the conclusions supported by the data presented?

-Are the limitations of analysis clearly described?

-Do the authors discuss how these data can be helpful to advance our understanding of the topic under study?

-Is public health relevance addressed?

Reviewer #1: yes

Reviewer #2: The conclusions are generally supported by the data presented and appropriately emphasize the limited and heterogeneous evidence base. The public health relevance of the topic is clear, particularly given the scarcity of prevalence and burden estimates across the region.

However, the interpretation of the pooled prevalence estimate would benefit from additional contextual clarification. While the manuscript does not explicitly frame the pooled value as a regional prevalence, it would be helpful to clearly state that prevalence data are available from only a subset of countries within the region, with many countries not represented by any eligible studies. Explicitly acknowledging this incomplete geographical coverage would help readers better understand the scope and limitations of the pooled estimate.

In addition, substantial differences in prevalence across the included countries are evident and likely reflect meaningful epidemiological variation, potentially driven by differences in environmental exposure, population characteristics, and local contexts. Highlighting these aspects more clearly in the Discussion and Conclusions would further strengthen interpretation and public health relevance.

Reviewer #3: The discussion section can be shortened. The authors' main point is relevant, but they need to explain why podoconiosis is neglected among neglected tropical diseases. They should provide data on DALYs for other NTDs and podoconiosis, as well as funding amounts allocated. These points need to be better supported with data in both the introduction and the discussion. Additionally, the authors don’t address their findings in relation to the predictive model described in the introduction, which would be beneficial.

**********

**Editorial and Data Presentation Modifications?**

Use this section for editorial suggestions as well as relatively minor modifications of existing data that would enhance clarity. If the only modifications needed are minor and/or editorial, you may wish to recommend “Minor Revision” or “Accept”.

Reviewer #1: Minor Revision

Reviewer #2: Several editorial and data presentation refinements would enhance the clarity and readability of the manuscript without altering the underlying analyses.

Specifically, further visual standardization of the PRISMA flow diagram (Figure 1) would improve readability. Figure 2 would benefit from the addition of a clear legend to facilitate interpretation. In Figure 3 (forest plot), including references or unique study identifiers alongside study labels would allow readers to more easily identify individual studies. Figure 5 would also benefit from a clearer and more informative legend to better explain its content and purpose.

In addition, minor formatting improvements could be made to Table 1, such as standardizing the labeling of the “Author” column to ensure consistency throughout the table.

Finally, for transparency and reproducibility, providing the full search strategies used for each database, ideally as supplementary material, would be helpful and align the manuscript more closely with PRISMA 2020 reporting recommendations.

Reviewer #3: Figure 2, the number of studies can be shown on the map.

Figure 4: The dots in the funnel chart should be labelled with the studies.

Table 1 contains dates and timestamps in two rows that need to be removed.

The paragraphs in the introduction mentioning 57 million DALYs, 22 million DALYs, and the global burden of disease are very confusing. The authors need to rewrite it so that there is a clear narrative flow and logic leading to the point they are making.

**********

**Summary and General Comments**

Use this section to provide overall comments, discuss strengths/weaknesses of the study, novelty, significance, general execution and scholarship. You may also include additional comments for the author, including concerns about dual publication, research ethics, or publication ethics. If requesting major revision, please articulate the new experiments that are needed.

Reviewer #1: (No Response)

Reviewer #2: This manuscript addresses an important and under explored topic and represents a valuable attempt to synthesize the evidence accumulated over the past decade on the prevalence and burden of podoconiosis in East Africa. The topic is of clear public health relevance, given the limited surveillance data available and the growing need to better understand the epidemiology and burden of the disease in resource-limited settings. The authors should be commended for assembling a challenging and heterogeneous body of literature and for attempting a quantitative synthesis in a field characterized by sparse data.

The systematic review is generally well conducted, and the overall study design is appropriate. The use of a random-effects meta-analysis reflects an awareness of substantial between-study heterogeneity, and the manuscript highlights important gaps in the literature, particularly the scarcity of burden estimates based on DALYs. These elements contribute to the novelty and potential significance of the work.

However, several issues require major revision before the manuscript can be considered for publication. These concerns primarily relate to transparency, clarity, and interpretation rather than to the generation of new data. In particular, the search strategy and geographical scope require clearer specification to ensure reproducibility. It is also essential that the manuscript clearly reports when the literature search was last conducted and whether it was updated prior to submission, in order to allow readers to assess the currency and completeness of the evidence base.

Furthermore, the presentation and interpretation of the pooled prevalence estimate warrant additional refinement. Given the extreme heterogeneity across studies and substantial differences in epidemiological context between countries, the pooled estimate should be more clearly framed as a statistical summary rather than as a proxy for regional or population-level prevalence. Strengthening the Discussion and Conclusions to explicitly acknowledge these limitations is essential to avoid potential misinterpretation by readers.

Several aspects of data presentation and figure clarity also require improvement, including greater consistency between text, tables, and figures, clearer labeling of visual elements, and more informative legends. These revisions would substantially enhance readability and coherence without altering the underlying analyses.

Overall, the study is of interest and has the potential to make a meaningful contribution to the literature. With major revisions focused on methodological transparency, clearer reporting, and more cautious interpretation of the findings—particularly with respect to the pooled prevalence estimate and the currency of the evidence base—the manuscript would be significantly strengthened.

Reviewer #3: The manuscript titled “The Health and Economic Burden of Podoconiosis in East Africa: A Systematic Review and Meta-Analysis” is a systematic review in which the authors have screened over 900 articles. They provide pooled prevalence estimates using random effects from 14 studies. This is a fairly well-written paper. The inclusion of DALYs into the paper does not make sense since only one study is available. The main point about podoconiosis being neglected within the NTD is a valid observation. I have provided suggestions for the methods, results, and minor editorial issues above, which the authors need to incorporate.

**********

PLOS authors have the option to publish the peer review history of their article (what does this mean?). If published, this will include your full peer review and any attached files.

If you choose “no”, your identity will remain anonymous but your review may still be made public.

**Do you want your identity to be public for this peer review?**  For information about this choice, including consent withdrawal, please see our Privacy Policy.

Reviewer #1: **Yes:** Ok

Reviewer #2: No

Reviewer #3: No

[NOTE: If reviewer comments were submitted as an attachment file, they will be attached to this email and accessible via the submission site. Please log into your account, locate the manuscript record, and check for the action link "View Attachments". If this link does not appear, there are no attachment files.]

**Figure resubmission:**

While revising your submission, we strongly recommend that you use PLOS’s NAAS tool (https://ngplosjournals.pagemajik.ai/artanalysis) to test your figure files. NAAS can convert your figure files to the TIFF file type and meet basic requirements (such as print size, resolution), or provide you with a report on issues that do not meet our requirements and that NAAS cannot fix.

After uploading your figures to PLOS’s NAAS tool - https://ngplosjournals.pagemajik.ai/artanalysis, NAAS will process the files provided and display the results in the "Uploaded Files" section of the page as the processing is complete. If the uploaded figures meet our requirements (or NAAS is able to fix the files to meet our requirements), the figure will be marked as "fixed" above. If NAAS is unable to fix the files, a red "failed" label will appear above. When NAAS has confirmed that the figure files meet our requirements, please download the file via the download option, and include these NAAS processed figure files when submitting your revised manuscript.

**Reproducibility:**

To enhance the reproducibility of your results, we recommend that authors of applicable studies deposit laboratory protocols in protocols.io, where a protocol can be assigned its own identifier (DOI) such that it can be cited independently in the future. Additionally, PLOS ONE offers an option to publish peer-reviewed clinical study protocols. Read more information on sharing protocols at https://plos.org/protocols?utm_medium=editorial-email&utm_source=authorletters&utm_campaign=protocols

Attachment Submitted filename: Reviewers comments.docx

# Author comment: Author response to Decision Letter 1

**DOI:** 10.1371/journal.pntd.0014427.r002

13 Apr 2026

Attachment Submitted filename: Response_to_PLOS_Reviewers_Comments_SR_April112026.docx

# Peer review: Decision Letter 1

**DOI:** 10.1371/journal.pntd.0014427.r003

**Authors:** Maria Victoria Periago, Maria Victoria Periago

1 Jun 2026

Dear Miss Hitimana,

We are pleased to inform you that your manuscript 'The Health and Economic Burden of Podoconiosis in East Africa: A Systematic Review and Meta-Analysis of Health Outcomes with Narrative Synthesis of Economic Evidence' has been provisionally accepted for publication in PLOS Neglected Tropical Diseases.

Before your manuscript can be formally accepted you will need to complete some formatting changes, which you will receive in a follow up email. A member of our team will be in touch with a set of requests.

Please note that your manuscript will not be scheduled for publication until you have made the required changes, so a swift response is appreciated.

IMPORTANT: The editorial review process is now complete. PLOS will only permit corrections to spelling, formatting or significant scientific errors from this point onwards. Requests for major changes, or any which affect the scientific understanding of your work, will cause delays to the publication date of your manuscript.

Should you, your institution's press office or the journal office choose to press release your paper, you will automatically be opted out of early publication. We ask that you notify us now if you or your institution is planning to press release the article. All press must be co-ordinated with PLOS.

Thank you again for supporting Open Access publishing; we are looking forward to publishing your work in PLOS Neglected Tropical Diseases.

Best regards,

Maria Victoria Periago

Section Editor

PLOS Neglected Tropical Diseases

Maria Victoria Periago

Section Editor

PLOS Neglected Tropical Diseases

Shaden Kamhawi

co-Editor-in-Chief

PLOS Neglected Tropical Diseases

orcid.org/0000-0003-4304-636XX

Paul Brindley

co-Editor-in-Chief

PLOS Neglected Tropical Diseases

orcid.org/0000-0003-1765-0002

***********************************************************

Reviewer's Responses to Questions

**Key Review Criteria Required for Acceptance?**

As you describe the new analyses required for acceptance, please consider the following:

**Methods**

-Are the objectives of the study clearly articulated with a clear testable hypothesis stated?

-Is the study design appropriate to address the stated objectives?

-Is the population clearly described and appropriate for the hypothesis being tested?

-Is the sample size sufficient to ensure adequate power to address the hypothesis being tested?

-Were correct statistical analysis used to support conclusions?

-Are there concerns about ethical or regulatory requirements being met?

Reviewer #3: This is a systematic review paper. The authors have followed standard protocols for conducting the review. The pooled prevalence has been estimated using appropriate methods. The artcile selection and integration into analysis are appropriate.

**********

**Results**

-Does the analysis presented match the analysis plan?

-Are the results clearly and completely presented?

-Are the figures (Tables, Images) of sufficient quality for clarity?

Reviewer #3: The results have been presented in a lucid and cogent way. The subanalysis of Ethiopia, as per my earlier comments, has been incorporated. All the mistakes in the previous versions have been rectified. The concerns I had raised have been addressed, and the suggestions I had made have been incorporated. The authors need to seperate results from discussion. They have merged teh two sections for each of their findings.

**********

**Conclusions**

-Are the conclusions supported by the data presented?

-Are the limitations of analysis clearly described?

-Do the authors discuss how these data can be helpful to advance our understanding of the topic under study?

-Is public health relevance addressed?

Reviewer #3: The conclusions are supported by the meta-analysis. The strengths and limitations are described.

**********

**Editorial and Data Presentation Modifications?**

<br/>

Use this section for editorial suggestions as well as relatively minor modifications of existing data that would enhance clarity. If the only modifications needed are minor and/or editorial, you may wish to recommend “Minor Revision” or “Accept”.

Reviewer #3: One paragraph on page 24 beginning "Given the dominance of Ethiopian studies in the overall evidence base and the substantial between-country heterogeneity.... " is repeated and needs to be removed. The results section has some informal language which can be removed like the use of phrases

funnel plot asymmetry is therefore more plausibly explained by genuine between-study heterogeneity arising . The authors need to explain in the methods the additional analysis done , and present the findings in the results. The authors seem to be mixing both in the results.

**********

**Summary and General Comments**

Use this section to provide overall comments, discuss strengths/weaknesses of the study, novelty, significance, general execution and scholarship. You may also include additional comments for the author, including concerns about dual publication, research ethics, or publication ethics. If requesting major revision, please articulate the new experiments that are needed.

Reviewer #3: The overall paper is good, given the paucity of prevalence studies. It not only highlights the gap in prevalence estimation but also how to move ahead for conducting rigorous prevalence studies in future.

**********

PLOS authors have the option to publish the peer review history of their article (what does this mean?). If published, this will include your full peer review and any attached files.

If you choose “no”, your identity will remain anonymous but your review may still be made public.

**Do you want your identity to be public for this peer review?**  For information about this choice, including consent withdrawal, please see our Privacy Policy.

Reviewer #3: No

# Editor report: Acceptance letter

**DOI:** 10.1371/journal.pntd.0014427.r004

**Authors:** Maria Victoria Periago, Maria Victoria Periago

Dear Miss Hitimana,

We are delighted to inform you that your manuscript, "The Health and Economic Burden of Podoconiosis in East Africa: A Systematic Review and Meta-Analysis of Health Outcomes with Narrative Synthesis of Economic Evidence," has been formally accepted for publication in PLOS Neglected Tropical Diseases.

We have now passed your article onto the PLOS Production Department who will complete the rest of the publication process. All authors will receive a confirmation email upon publication.

The corresponding author will soon be receiving a typeset proof for review, to ensure errors have not been introduced during production. Please review the PDF proof of your manuscript carefully, as this is the last chance to correct any scientific or type-setting errors. Please note that major changes, or those which affect the scientific understanding of the work, will likely cause delays to the publication date of your manuscript. Note: Proofs for Front Matter articles (Editorial, Viewpoint, Symposium, Review, etc...) are generated on a different schedule and may not be made available as quickly.

Soon after your final files are uploaded, the early version of your manuscript will be published online unless you opted out of this process. The date of the early version will be your article's publication date. The final article will be published to the same URL, and all versions of the paper will be accessible to readers.

For Research Articles, you will receive an invoice from PLOS for your publication fee after your manuscript has reached the completed accept phase. If you receive an email requesting payment before acceptance or for any other service, this may be a phishing scheme. Learn how to identify phishing emails and protect your accounts at https://explore.plos.org/phishing.

Thank you again for supporting open-access publishing; we are looking forward to publishing your work in PLOS Neglected Tropical Diseases.

Best regards,

Shaden Kamhawi

co-Editor-in-Chief

PLOS Neglected Tropical Diseases

Paul Brindley

co-Editor-in-Chief

PLOS Neglected Tropical Diseases
